# Hydrogen Isotope Separation Using a Metal–Organic Cage Built from Macrocycles

**DOI:** 10.1002/anie.202202450

**Published:** 2022-07-04

**Authors:** Donglin He, Linda Zhang, Tao Liu, Rob Clowes, Marc A. Little, Ming Liu, Michael Hirscher, Andrew I. Cooper

**Affiliations:** ^1^ Materials Innovation Factory and Department of Chemistry University of Liverpool 51 Oxford Street Liverpool L7 3NY UK; ^2^ Max Planck Institute for Intelligent Systems Heisenbergstr. 3 70569 Stuttgart Germany; ^3^ Department of Chemistry Zhejiang University Hangzhou 310027 China; ^4^ ZJU-Hangzhou Global Scientific and Technological Innovation Center Hangzhou 311215 China; ^5^ Leverhulme Research Centre for Functional Materials Design University of Liverpool 51 Oxford Street Liverpool L7 3NY UK

**Keywords:** Hydrogen Isotope Separation, Macrocycles, Metal–Organic Cages, Porous Materials, Self-Assembly

## Abstract

Porous materials that contain ultrafine pore apertures can separate hydrogen isotopes via kinetic quantum sieving (KQS). However, it is challenging to design materials with suitably narrow pores for KQS that also show good adsorption capacities and operate at practical temperatures. Here, we investigate a metal–organic cage (MOC) assembled from organic macrocycles and Zn^II^ ions that exhibits narrow windows (<3.0 Å). Two polymorphs, referred to as **2α** and **2β**, were observed. Both polymorphs exhibit D_2_/H_2_ selectivity in the temperature range 30–100 K. At higher temperature (77 K), the D_2_ adsorption capacity of **2β** increases to about 2.7 times that of **2α**, along with a reasonable D_2_/H_2_ selectivity. Gas sorption analysis and thermal desorption spectroscopy suggest a gate‐opening effect of the MOCs pore aperture. This promotes KQS at temperatures above liquid nitrogen temperature, indicating that MOCs hold promise for hydrogen isotope separation in real industrial environments.

## Graphical abstract

Metal–organic cages (MOCs), built from macrocycles, were investigated for the first time for the separation of hydrogen isotopes. The MOC crystal has flexible narrow pores, which leads to a separation temperature of over 100 K even without existing strong adsorption sites.

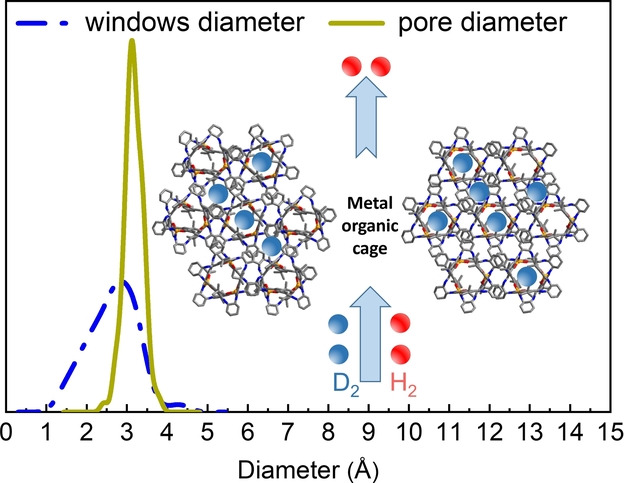

## Introduction

Deuterium (D_2_) is a crucial fuel for future fusion power plants. D_2_ is also used as a neutron moderator,^[1]^ in neutron scattering experiments,^[2]^ and as a nonradiative isotope tracer.^[3]^ These applications require high purity D_2_, which is non‐trivial because of its low natural abundance of 0.0156 mol %. Typically, D_2_ is purified industrially using the Girdler sulfide process^[4]^ or by cryogenic distillation.^[5]^ However, both methods are inefficient and energy‐intensive.[^1a^, ^3b^] An attractive alternative to separate D_2_ from its dominant isotope, hydrogen, is to adsorb D_2_ selectively on a microporous bed.

Kinetic quantum sieving (KQS), first proposed by Beenakker *et al*.,^[6]^ describes the effect of lighter isotopes with larger de Broglie wavelengths encountering higher energy barriers as they diffuse through fine pores at cryogenic temperatures. KQS effects lead to differences between the diffusion rates of isotopes, making it a potential process for the separation of D_2_ from H_2_. KQS requires adsorbents with ultrafine pore apertures; typically <5 Å,^[7]^ with pore apertures of 3.4 Å reported as the optimal size in rigid frameworks under cryogenic conditions.^[8]^ Owing to their small pore sizes, several microporous materials have been investigated for KQS of hydrogen isotopes, including porous carbons,^[9]^ zeolites,^[10]^ metal‐organic frameworks,^[11]^ and covalent organic frameworks.^[8]^ We also recently reported a porous organic cage (POC) co‐crystal that combined cages with narrow pores and cages with good capacities to achieve optimal separation performance in KQS.^[12]^ However, despite some recent success, it remains challenging to precisely tune the pore size to the desired level required for KQS without compromising the adsorption capacity of the material. Also, the best selectivities tend to be achieved at very low temperatures (30–40 K), which is energetically costly.

Metal–organic cages (MOCs),^[13]^ also known as metal–organic polygons or polyhedrons (MOPs),^[14]^ are discrete molecules with intrinsic cavities, formed from metal cations and organic linkers. Like organic cages, many MOCs have good solubility in a range of solvents and thus can be processed into different forms to optimize their structures and functions. MOCs can also contain open metal sites, which can enhance their gas adsorption properties.^[15]^ To date, MOCs have been demonstrated to selectively adsorb CO_2_,^[16]^ O_2_,^[17]^ CO,^[18]^ NO,^[19]^ and C_3_H_8_.^[20]^ due to their specific pore sizes or open metal sites. With small pores that could be suitable for KQS and, potentially, open metal sites to enhance adsorption affinity, MOCs are interesting candidates for hydrogen isotope separation.[^15c^, ^21^]

The solid‐state porosity of MOCs is affected by guest accessibility to the intrinsic MOC cavity and extrinsic porosity in their structures.^[22]^ The intrinsic porosity of MOCs can be controlled by choosing appropriate organic linkers and metal centres,^[23]^ or by post‐synthetic modification.^[24]^ The extrinsic porosity in MOC solids can be controlled, to an extent, by using crystal engineering methods.^[25]^ An important consideration is that the organic units (or ligands) are key structural components of MOCs and this significantly affects their molecular flexibility and porosity.[^19^, ^26^] Although linear or planar organic linkers are the most common building units for MOCs, macrocycles with intrinsic voids or cavities, such as porphyrins^[27]^ calixarene,^[19]^ and calixsalens^[28]^ have emerged recently as alternative MOCs building units. With their own intrinsic prefabricated cavities, macrocycles can enrich the functionality and structural diversity of MOCs. For example, calixarene‐based macrocycles have been coordinated to tetranuclear clusters to form permanently porous MOCs^[29]^ with surface areas as high as 1239 m^2^ g^−1^.^[19]^

**Figure 1:**
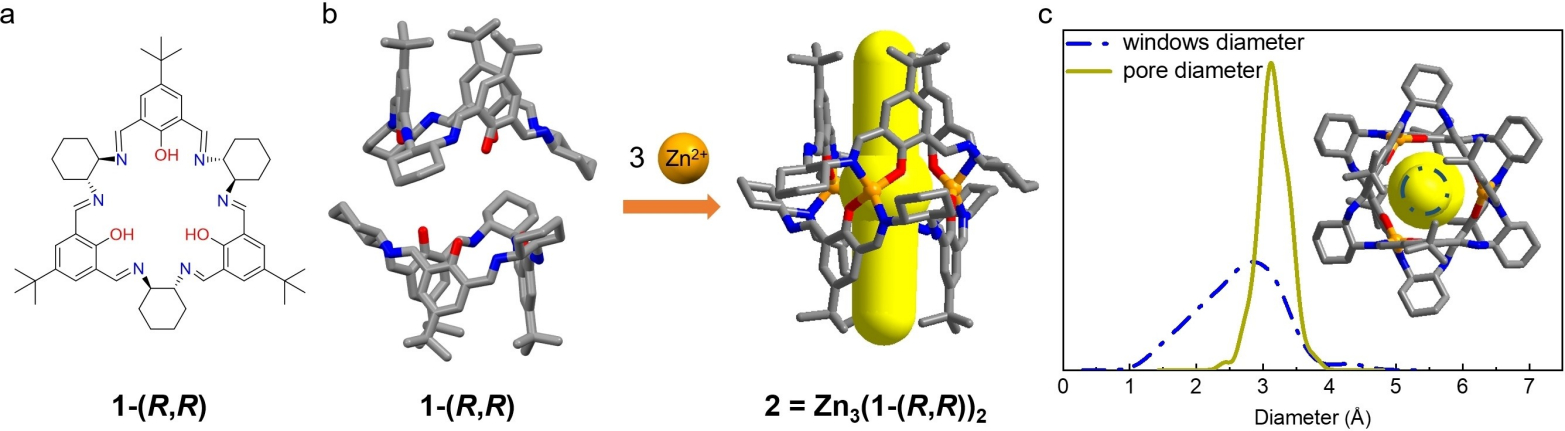
a) Structure of macrocycle **1**‐(*R*,*R*). b) The self‐assembly of **2** from macrocycle **1**‐(*R*,*R*) using Zn^II^ ions. c) Window diameter and pore diameter distribution histograms of **2**. The window diameter and pore diameter distribution histograms share the bottom X‐axis. Atom colours: Zn, orange; N, blue; O, red; C, grey.

Calixsalen macrocycles have a bowl‐like shape and small intrinsic cavity (Figure 1). They were first reported in 1999 by Jablonski et al., who condensed 2‐hydroxyisophthalaldehyde derivatives with chiral (1*R*,2*R*)‐cyclohexane‐1,2‐diamine.^[30]^ In 2018, Barbour *et al*. found that calixsalens with Cl and Br substituents displayed remarkable sorption properties for ethylene and carbon dioxide, which suggested the potential of adjusting their sorption properties by introducing functional groups.^[30b]^ As shown in Figure 1b, the enantiopure [3+3] calixsalen macrocycle, **1**‐(*R*,*R*), has a rim and a tail. The rim consists of three cyclohexane rings and three hydroxy‐substituted aromatic rings, while the tail consists of three bulky *tert*‐butyl groups.^[31]^ In the structure of **1**‐(*R*,*R*), the three salen units have the same orientation,^[31]^ and coordinating **1**‐(*R*,*R*) to metal ions, including Zn^II^, has been used previously by Lisowski *et al*. to connect molecules of **1**‐(*R*,*R*) and form the trinuclear Zn MOC, **2** (Figure 1b).^[28]^ The same team also reported that **2** has a Brunauer–Emmett–Teller surface area (SA_BET_) of 610 m^2^ g^−1^ after being activated at ambient temperature, and this material can enantioselectively bind chiral alcohols.^[32]^ Most recently, **2** has been used for gas chromatographic separation,^[33]^ and chiral separation.^[34]^

The previously reported solvated single crystal structure of **2** shows that each MOC has a hollow cavity in a tubular shape with two narrow windows at both ends.^[32]^ As shown in Figure 1c, the window diameter and pore diameter distribution histograms were calculated by py‐window^[35]^ based on xTB^[36]^ MD trajectory of **2** (see Supporting Information Section 1.5.8). The pore diameter of **2** is around 3.1 Å. In addition, the broad window diameter distribution below 2 Å is due to the rotation of the three *tert*‐butyl groups. Furthermore, the window diameter of around 2.8 Å suggests that **2** has the optimal diameter for KQS.^[12]^ Here, we investigated the hydrogen isotope separation performance of MOCs for the first time. We successfully obtained the single‐crystal structures of the two activated polymorphs of **2**, referred to here as **2α** and **2β**. We found there are slight differences in the activated crystal structures between the orientation of *tert*‐butyl groups and the crystal packing of the MOCs. These structural differences led to contrasting D_2_/H_2_ separation performance, which was evaluated by thermal desorption spectroscopy (TDS).

## Results and Discussion

**Figure 2:**
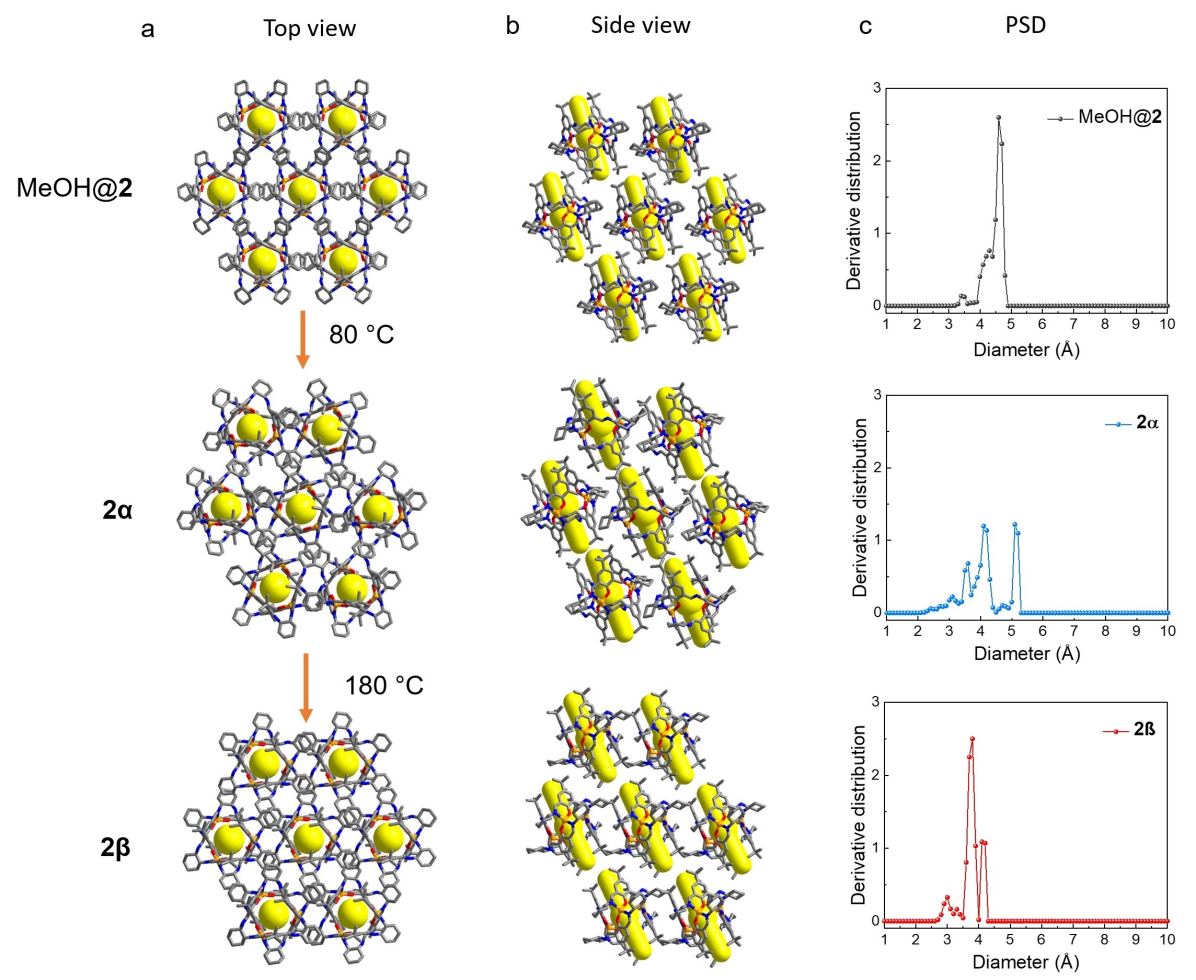
Crystal packing images of MeOH@**2** (top, methanol solvent is omitted for clarity), **2α** (middle) and **2β** (bottom) in a) top view and b) side view. H atoms are omitted for clarity (orange, Zn; blue, N; red, O; grey, C). The intrinsic cavity is highlighted in yellow. c) PSD (pore size distributions) histograms for MeOH@**2** (black), **2α** (blue,) and **2β** (red).

The [3+3] calixsalene macrocycle, **1**‐(*R*,*R*), shown in Figure 1a, is synthesized by reacting (1*R*,2*R*)‐cyclohexane‐1,2‐diamine with 4‐*tert*‐butyl‐2,6‐diformaylphenol.[^32^, ^37^] Reacting **1**‐(*R*,*R*) with Zn^II^ acetate in a 2 : 3 ratio in methanol affords **2**, which comprises two triply deprotonated macrocycles **1**‐(*R*,*R*)^3−^ held together by three Zn^II^ ions in a sandwich‐like conformation (Figure 1b).[^28^, ^32^] Solvated single crystals of **2** with monoclinic *Cc* space group symmetry, referred to as MeOH@**2**, are obtained as large yellow block crystals from the reaction solvent,[^32^, ^37^] (Figure 2a and Table S1) and a previous study reported that activated crystals of **2** were unsuitable for single‐crystal X‐ray diffraction analysis.^[32]^ However, in this study, we successfully obtained two solvent‐free single‐crystal structures of **2** after thermally activating high‐quality crystals of MeOH@**2**, grown in the reaction solvent at room temperature over 12 hours. To activate MeOH@**2**, we collected the solvated crystals by filtration and heated the sample under a vacuum at 80 °C. Thermogravimetric analysis (TGA) and NMR were used to confirm that the crystals were fully activated (Figure S2, S6). Removing the reaction solvent at 80 °C under vacuum induces MeOH@**2** to transform into a new phase, referred to as **2α**, which has a different crystal packing of MOCs and lower triclinic *P*1 space group symmetry (Figure 2a and see Table S1). Further heating of **2α** at 180 °C under vacuum for 12 hours yielded another new polymorph, **2β** (Figure 2a), again with triclinic *P*1 space group symmetry (see Table S1). We used differential scanning calorimetry (DSC) and in situ variable‐temperature powder X‐ray diffraction analysis (PXRD) to monitor the transformation between **2α** to **2β** (Figure S7, S8b). As shown in Figure S7, the DSC curve of **2α** shows an endotherm between 168 °C to 183 °C, which we attribute to the transformation from **2α** to **2β**. By contrast, the DSC of **2β** did not show any endotherms over this temperature range, suggesting **2β** is the more thermostable phase. Furthermore, in situ variable‐temperature PXRD data (Figure. S8b) indicates that **2α** is stable between room temperature and 125 °C, but at 160 °C begins to transform. At 180 °C, the PXRD pattern closely matched the simulated PXRD of **2β**, which is consistent with the DSC curve of **2α**.

As shown in Figure 2a and 2b, **2** in MeOH@**2**, **2α** and **2β** has a tubular‐shaped intrinsic cavity shape, highlighted using a yellow cylinder, with a wider cavity at the centre, highlighted using a yellow sphere. In MeOH@**2**, there are also large solvent‐filled extrinsic voids between aligned molecules of **2**. After thermally removing solvent from MeOH@**2** at 80 °C under vacuum to form **2α**, the tubular‐shaped MOC cavities are preserved, but the MOCs pack more closely, and the extrinsic voids are smaller. For comparison, after deleting the solvent from MeOH@**2**, the solvent‐accessible volume calculated by Platon using a probe radii of 1.2 Å accounts for about 36.9 % of the unit cell volume. By contrast, the comparable void volume in **2α** is 17.3 % of the unit cell volume. In **2β**, which was obtained by heating **2α** at 180 °C under vacuum, the void volume increased slightly to about 20.6 % of unit cell volume. This difference is mainly due to the crystal packing of **2** because the molecular overlay plot of **2α** (blue) and **2β** (red) shown in Figure S20 indicates that the molecular structures of **2** in **2α** and **2β** are very similar. In addition, we used Zeo++^[38]^ to compare the pore structures in MeOH@**2**, **2α**, and in more detail and calculate their pore size distributions (PSD, see Supporting Information Section 1.5.8 for full details). These calculations were performed after removing the solvent, including residual water, from the crystal structures. The largest free spheres (D_f_) in MeOH@**2**, **2α**, and **2β**, calculated by Zeo++ are 3.4, 2.0 and 2.6 Å, respectively, and these values represent their pore limiting diameters.^[39]^ The PSD plot of MeOH@**2** shows three peaks at 3.4 Å, 4.3 Å and 4.6 Å (Figure 2c). In the PSD plots of **2α** and **2β**, there are also three peaks, but the centres vary from 3.6, 4.1, and 5.1 Å in **2α** to 3.0 Å, 3.8 Å and 4.1 Å in **2β**. Therefore, the pore sizes of **2α** and **2β** are around 2.0 to 5.1 Å and 2.6 to 4.1 Å, respectively, satisfy the requirement of porous materials for KQS applications.

**Figure 3:**
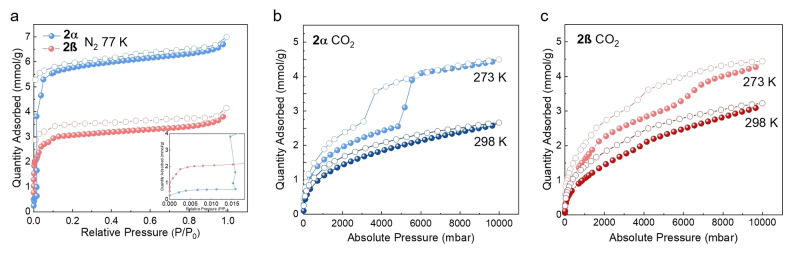
a) N_2_ sorption isotherms at 77 K for **2α** (blue) and **2β** (red). b) CO_2_ sorption isotherms at 273 and 298 K for **2α**. c) CO_2_ sorption isotherms at 273 K and 298 K for **2β**. Solid symbols: adsorption; hollow symbols: desorption.

To quantify how the PSD of **2α** and **2β** affected their porosity, we measured their N_2_, Ar, CO_2_ and H_2_ gas adsorption isotherms. As shown in Figure 3a, **2α** only adsorbed a small amount of N_2_ (<0.6 mmol g^−1^) below *P*/*P*_0_=0.015. However, after reaching a *P*/*P*_0_ of 0.015, we observed a step in the adsorption isotherm, and the N_2_ uptake increased sharply to >5 mmol g^−1^. This result is consistent with the pressure‐induced gating effect previously reported for **2**.^[32]^ By contrast, **2β** has a Type‐I N_2_ isotherm at 77 K (Figure 3a), and in the absence of pressure‐induced gating effect observed for **2α**, had the lower uptake at 1 bar (7.0 mmol g^−1^ for **2α** vs. 4.2 mmol g^−1^ for **2β**) and calculated SA_BET_ (394 m^2^ g^−1^ for **2α** vs. 270 m^2^ g^−1^ for **2β**). Hence, even though the calculated void volume in **2β** was higher than **2α** (17.3 % for **2α** vs. 20.6 % for **2β**), the pressure‐induced gating effect appears to have created additional porosity in **2α**.

To further investigate the flexibility of the pore and the void volume for the gas sorption, higher pressure CO_2_ and H_2_ isotherms were measured for **2α** and **2β** from 0 to 10 bar. As shown in Figure 3b and 3c, two successive plateaus in the high‐pressure CO_2_ isotherm of **2α** and **2β** at 273 K indicate a pressure‐induced gating effect,^[40]^ but this effect appeared more profound for **2α** than **2β**. At 298 K, the CO_2_ isotherm hysteresis was less extensive for **2α** and **2β**. A pronounced hysteresis loop was found in the H_2_ isotherms of **2α** at 77 K from 0 to 10 bar. By contrast, no distinct hysteresis loop was observed in the H_2_ isotherms of **2β** (Figure S14). In conclusion, the higher‐pressure isotherms are consistent with the N_2_ isotherms, which suggest that **2α** is more structurally flexible than **2β**.

**Figure 4:**
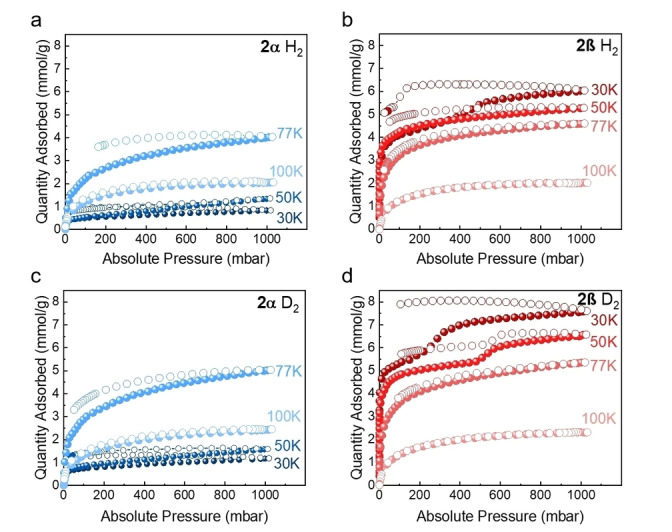
H_2_ isotherms of a) **2α** and b) **2β**; D_2_ isotherms of c) **2α** and d) **2β** recorded at 30 K, 50 K, 77 K and 100 K. Solid symbols: adsorption; hollow symbols: desorption.

H_2_ and D_2_ gas sorption isotherms for **2α** and **2β** recorded at various temperatures (30, 50, 77, and 100 K) are shown in Figure 4. **2α** has low D_2_ and H_2_ gas uptakes at 30 and 50 K (≈1 mmol g^−1^ or lower at 1 bar), but much higher uptakes of 3.8 mmol g^−1^ for H_2_ and 4.9 mmol g^−1^ for D_2_ at 77 K and 1 bar. The increased H_2_ and D_2_ uptakes at the higher temperatures, where surface adsorption effects are also likely to be less profound, indicate that D_2_ and H_2_ can more easily penetrate the MOC cavities at 77 K. In addition, the **2α** isotherms exhibit hysteresis, which is less pronounced at 100 K, implying faster equilibration at higher temperatures. The stronger hysteresis at lower temperature, which decreases at higher temperatures, denotes a temperature‐dependent diffusion limitation of gas molecules penetrating the cavities through different aperture sizes. Unlike typical isotherms that feature lower uptakes at increased temperature, **2α** has lower H_2_ and D_2_ uptakes at 30 and 50 K compared to that observed at 77 K. This can be ascribed to a very narrow pore aperture at low temperature, which prohibits gas molecules from diffusing deeply into the **2α** crystals. The gas uptakes, therefore, increase with higher temperature, denoting a larger pore aperture that is thermally opened due to the thermal vibration of the flexible windows. By contrast, the D_2_ and H_2_ adsorption isotherms for **2β** reached a maximum uptake of 7.5 mmol g^−1^ for D_2_ and 5.9 mmol g^−1^ for H_2_ at 30 K and 1 bar (Figure 4b and d), indicating that this MOC crystal is fully accessible to these gases even at lower temperatures. There was again strong hysteresis at 30 K, but again this hysteresis was reduced with increasing temperature, indicating better equilibrium. For **2α** and **2β**, we observed higher D_2_ uptakes than H_2_ at all measurement temperatures, which we attribute to higher diffusion rates and increased heats of adsorption for D_2_. The D_2_ gas sorption isotherms show that **2β** has a two‐step H_2_ adsorption isotherm at 30 K. Two‐step D_2_ adsorption isotherms for **2β** were also observed at 30 and 50 K. The stepped isotherms can be attributed to the phase transition of the adsorbate. Similar phenomena have been observed before: for example, a step in the low‐pressure region of a MOF (MET‐2) Ar and N_2_ isotherms has been reported by Gándara et al., which was attributed to the phase transition of the adsorbate.^[41]^ This suggests that **2β** can accommodate extra gas molecules after all the initially accessible adsorption sites are fully occupied. For **2β**, when the pores are saturated with 5 mmol g^−1^ of H_2_ or 6 mmol g^−1^ of D_2_, further dosing of gases appears to open additional adsorption sites as the gas pressure increases. We used PXRD to confirm that the crystal structures of **2α** and **2β** did not change profoundly after the measurements. (Figure S10) The PXRD data show that the **2α** and **2β** remained in the same phase and were crystalline after H_2_ and D_2_ isotherms. Variable‐temperature PXRD also confirmed that **2α** and **2β** are stable between 30 to 100 K. (Figure S9) Hence, the stepped isotherms are due to local flexibility rather than more profound structural changes. Even though it has a similar structure, pore size, and accessible pore volume, similar steps were not observed in **2α**. We note that the structure of a physisorbed layer is dependent not only on the adsorbate‐adsorbate interactions but also on the magnitude and disposition of the adsorbent‐adsorbate interactions.^[41a]^ This difference can be ascribed to the distinctly different flexibility of **2α** and **2β** at very low temperatures (30 and 50 K), leading to a different accessible pore aperture.

Encouraged by the apparent faster D_2_ diffusion kinetics, we evaluated the ability of **2α** and **2β** to perform D_2_/H_2_ separations using a laboratory‐designed cryogenic thermal‐desorption spectroscope (TDS). First, we used pure H_2_ and D_2_ atmospheres in the TDS experiments to determine the preferred H_2_ and D_2_ adsorption sites in **2α** and **2β**. In these TDS measurements, **2α** and **2β** samples were exposed at room temperature to 10 and 200 mbar of pure H_2_ and D_2_, then cooled to 20 K under a gas atmosphere, and finally, the sample chamber was evacuated at 20 K. The TDS spectra were recorded while heating the samples from 20 and 170 K (Figure S19). The TDS spectra of **2α** (Figure S19a) collected after gas loading at 10 mbar shows little gas uptake. By contrast, the **2α** TDS spectra obtained from the 200 mbar gas loading shows one major peak for both isotopes, centered at about 102 K for D_2_ and 106 K for H_2_, with shoulders appearing at desorption temperatures below 70 K. The presence of the single peak with a shoulder shows there are at least two adsorption sites for H_2_ and D_2_ in **2α** with different adsorption potentials. The increase in gas uptake of both isotopes with higher pressure indicates the inner structure becomes more accessible at 200 mbar than 10 mbar. The TDS spectra for **2β** after gas loading at 10 mbar of H_2_ and D_2_ (Figure S19b) show small peaks at around 25 K and more profound peaks at around 90 K. In addition, two shoulders were observed over the temperature range 40–80 K. The TDS spectra of **2β** after gas loading at 200 mbar have one major peak at lower desorption temperatures; 86 K for D_2_ and 92 K for H_2_, compared to values of 102 K for D_2_ and 106 K for H_2_ observed for **2α**. We attribute the first desorption peak at low temperature (approximately 25 K) to weakly adsorbed gas molecules on the outer surfaces of the crystals. For both **2α** and **2β**, the temperature of the maximum desorption peak is lower for D_2_ than H_2_, denoting a faster diffusion of D_2_, which is in good agreement with H_2_ and D_2_ isotherms.

In the TDS spectra, the area under the desorption peak is proportional to the quantity of desorbed gas, which can be quantified by calibrating the mass spectrometer using a Pd_95_Ce_5_ alloy (see Supporting Information Section 1.5.7). At 200 mbar, the pure H_2_ and D_2_ uptakes for **2α** are 1.2 and 2.1 mmol g^−1^, respectively. By contrast, the pure H_2_ and D_2_ uptakes 200 mbar for **2β** are higher and were 2.4 and 3.8 mmol g^−1^, respectively, in good agreement with gas sorption isotherms.

**Figure 5:**
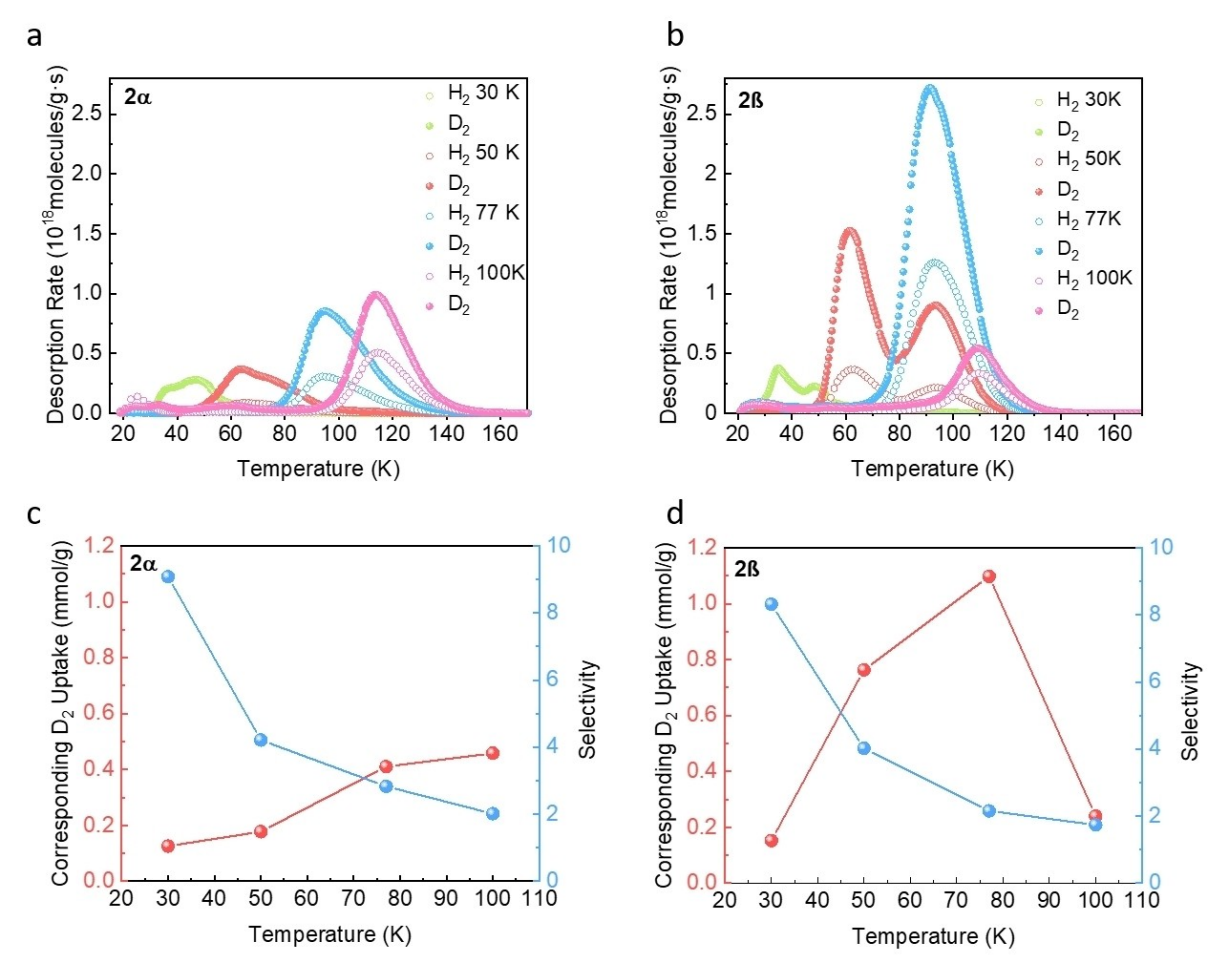
H_2_ (open) and D_2_ (close) thermal desorption spectra of 200 mbar 1 : 1 H_2_/D_2_ isotope mixture on a) **2α** and b) **2β** at different exposure temperatures, 30 K (green), 50 K (red), 77 K (blue), and 100 K (magenta). D_2_/H_2_ selectivity (blue) and the corresponding D_2_ uptake (red) as function of exposure temperature for c) **2α** and d) **2β**.

**Figure 6:**
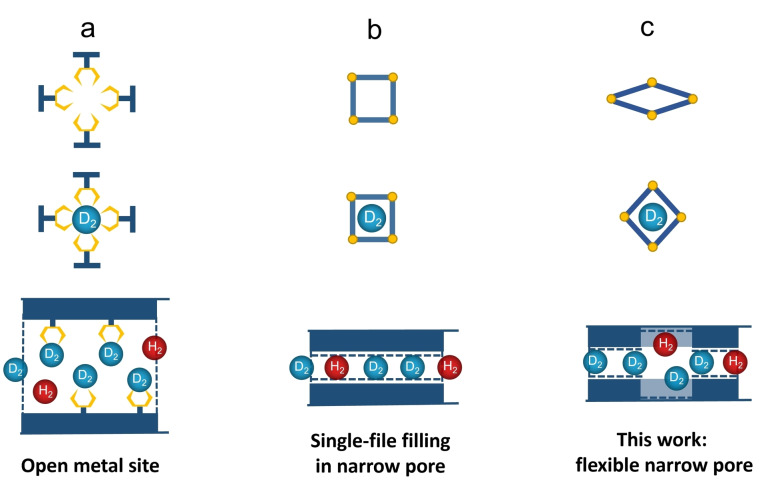
Three types of structures that could increase the practical temperature of hydrogen isotopes separation: a) Open metal sites.^[21a]^ b) Single‐file filling in ultrafine 1D channels.^[42]^ c) This work: flexible narrow pores.

We next used the TDS measurements to verify the competitive separation performances of **2α** and **2β**. These measurements were performed after directly exposing **2α** and **2β** to a 1 : 1 H_2_ : D_2_ mixture (200 mbar) for 10 min at exposure temperatures (*T*_exp_) between 30 and 100 K. The D_2_/H_2_ selectivity is then calculated from the ratio of the peak areas. The TDS spectra of **2α** are shown in Figure 5a, and Figure 5c shows the D_2_/H_2_ selectivity alongside the corresponding D_2_ uptake as a function of exposure temperature. The TDS spectra start from *T*_exp_ and measure the remaining free gas molecules released during the evacuation processes that are carried out at the same temperature. The gas uptakes increase with increasing temperatures, exhibiting a maximum of 100 K. Meanwhile, the selectivity decreases with increasing *T*_exp_, exhibiting the highest S${}_{D{}_{2}/H{}_{2}}$ =9.1 at 30 K. Generally, the strongest adsorption site, which corresponds to the highest desorption temperature, is occupied first and at very low loadings. The weaker sites are then occupied at higher gas loadings and this results in additional low‐temperature desorption peaks. However, the H_2_ and D_2_ TDS spectra of **2α** vary in shape and magnitude depending on *T*_exp_, which is contrary to the typical sequential filling behavior of accessible sites with different binding strengths. No desorption peak can be observed above 60 K for *T*_exp_=30 K, implying no deep penetration into the structure at this temperature. With increasing exposure temperature, gases can penetrate deeper into the crystals, and the desorption peaks in TDS spectra shift to higher temperatures. The gas molecules can finally penetrate the MOC crystals at *T*_exp_=100 K. These temperature‐dependent TDS spectra agree with the observation from pure gas isotherms, which is related to the temperature‐dependent gate‐opening behaviour.^[11b]^ In contrast, **2β** shows different desorption spectra under identical conditions, as shown in Figure 5b. For **2β**, the desorption spectrum at 30 K shows no desorption of any isotopes occurring above 50 K, indicating the weak adsorption of gas molecules on top of the surface. However, the TDS spectra measured at 50 K exhibit two desorption maxima that last until 120 K, indicating the gas molecules can freely access the crystal pores at 50 K. The D_2_/H_2_ selectivity and its corresponding D_2_ uptake as a function of exposure temperature are shown in Figure 5d, in which the highest S${}_{D{}_{2}/H{}_{2}}$ of 8.3 is observed at *T*_exp_=30 K. There is no considerable difference in the selectivity of **2α** and **2β** for D_2_/H_2_ (Figure 5c, d) from 30 K to 100 K. However, the D_2_ uptake for **2α** only slowly increases with increasing temperature, whereas the D_2_ for **2β** is far higher at 1.1 mmol g^−1^ at 77 K. The difference can be ascribed to a higher temperature for the opening of the pore aperture of **2α**, in which the exposed gas penetration is still limited at 77 K and needs a higher temperature for opening. The gas uptake in **2α** thus reaches the maximum at 100 K. By contrast, the aperture of **2β** fully opens at 77 K, allowing the gas molecules to be removed during the evacuation at exposure temperature prior to the TDS run. In addition, **2β** has a higher D_2_ uptake maximum than **2α**. The increasing isotope uptake with decreasing selectivity is related to the opening of the aperture and the sufficient kinetic energy of the molecule, where the accessibility of both isotopes is enhanced. Despite low selectivity, after exposure at *T*_exp_=100 K to an isotope mixture, the desorption maxima are centered at 115 K for both **2α** and **2β**, which is an unusual case exhibiting such high desorption temperature without the presence of strong adsorption sites. Therefore, both **2α** (S${}_{D{}_{2}/H{}_{2}}$ =2.8) and **2β** (S${}_{D{}_{2}/H{}_{2}}$ =2.2) have reasonable selectivity for D_2_ at 77 K compared with other porous materials without open metal sites. (Table S2) Generally, desorption above liquid N_2_ temperatures indicates the existence of strong binding sites, such as uncoordinated metals (Figure 6a).^[21a]^ However, the lack of open metal sites in MOCs limits the interaction with hydrogen molecules. A similar phenomenon has been reported by Mondal *et al*.^[42]^ in a series of I−D channel ultra‐microporous MOFs, called Imidazolate Framework Potsdam (IFP), which possess smaller pore size than the kinetic diameter of hydrogen molecules, which gets accessible to hydrogen by a temperature‐dependent dynamic gate opening. IFP‐4 and IFP‐7 exhibit a selectivity around 2 above liquid N_2_ temperature, which can be ascribed to KQS occurring only at the outermost pore aperture, since after penetrating the narrow pore channels, no passing of gas molecules is possible, and a single‐file filling occurs (Figure 6b). Additionally, the gas uptake at 77 K in IFP only reaches 0.01 mmol g^−1^ in IFP‐4 and 0.05 mmol g^−1^ in IFP‐7, due to the narrow I−D channels. By contrast, the gas uptake is much larger for our MOCs (0.41 mmol g^−1^ in **2α** and 1.10 mmol g^−1^ in **2β** at 77 K). In contrast to the rigid 1D channels in the IFP series, the ultrafine narrow pores in MOC **2** allow single‐file filling initially, but the flexible pores are adaptable and become more accessible to the target gas with increasing temperature. When the system is cooled down to low temperatures under a gas atmosphere, the gas molecules are captured on the additional inner surface and can be released by heating up to this temperature again (Figure 6c). Therefore, by introducing local flexibility into the MOC system, the practical temperature for hydrogen isotope separation can be increased dramatically and become comparable to the temperatures reached by MOFs possessing open metal sites.

## Conclusion

We have investigated two new polymorphs of a trinuclear Zn MOC for D_2_/H_2_ separation. The two polymorphs were isolated by activating crystals of MeOH solvate of **2**, initially at 80 °C to afford **2α**, and then at 180 °C to transform **2α** into **2β**. Surprisingly, **2β** had a slightly larger extrinsic porosity than **2α**. There are other differences between the crystal structures, and **2α** has a more extensive range of pore sizes from about 2.0 Å to 5.1 Å and appeared more flexible in gas sorption measurements, which led to **2α** having a higher BET surface area of 393.8 m^2^ g^−1^ compared to **2β** (269.9 m^2^ g^−1^). **2β** has a slightly higher unit cell void volume than **2α** (20.6 % for **2β** vs. 17.3 % for **2α**), and **2β** thus has a higher pure D_2_ capacity of 3.8 mmol g^−1^ than the **2α** (2.1 mmol g^−1^) at the exposing pressure of 200 mbar. In addition, TDS measurements confirm that the D_2_ adsorption capacity with **2β** (1.10 mmol g^−1^, S${}_{D{}_{2}/H{}_{2}}$ =2.2) is higher than for **2α** (0.41 mmol g^−1^, S${}_{D{}_{2}/H{}_{2}}$ =2.8) at 77 K for 1 : 1 D_2_‐H_2_ mixture. Furthermore, the local flexibility of MOC crystals provides the additional accessible inner surface to increase the adsorption and separation of hydrogen isotopes inside the crystals via KQS. This leads to a desorption temperature of over 100 K even without existing strong adsorption sites. Hence, the selectivity and capacity of MOCs for hydrogen isotopes are sensitive to their pore size and flexibility. This study paves the way for hydrogen isotope separation above liquid nitrogen temperatures based on well‐defined pore structures and the flexibility of molecular materials.

## Conflict of interest

The authors declare no conflict of interest.

1

## Supporting information

As a service to our authors and readers, this journal provides supporting information supplied by the authors. Such materials are peer reviewed and may be re‐organized for online delivery, but are not copy‐edited or typeset. Technical support issues arising from supporting information (other than missing files) should be addressed to the authors.

Supporting Information

Supporting Information

Supporting Information

Supporting Information

## Data Availability

The data that support the findings of this study are available in the Supporting Information of this article.
