# Supplementary material for: Hydrogen Isotope Separation Using a Metal–Organic Cage Built from Macrocycles
Source: Angew Chem Int Ed Engl. 2022 Jul 4;61(32):e202202450. doi: 10.1002/anie.202202450 (PMC9400858; doi:10.1002/anie.202202450)
Supplement: Supplementary file 4 — Supporting Information [file ANIE-61-0-s003.pdf]

Angewandte Chemie
Eine Zeitschrift der Gesellschaft Deutscher Chemiker

Supporting Information

Hydrogen Isotope Separation Using a Metal–Organic Cage Built from Macrocycles

D. He, L. Zhang, T. Liu, R. Clowes, M. A. Little*, M. Liu*, M. Hirscher, A. I. Cooper**

1. Experimental Details

1.1. Materials

(1*R*,2*R*)-1,2-Diaminocyclohexane was purchased from Manchester Organics, UK. All other chemicals and solvents (HPLC grade) were purchased from Sigma-Aldrich and used as received.

1.2. Macrocycle 1-(*R,R*)

Macrocycle **1**-(*R,R*) was synthesized as described previously.[1] A solution of 4–tert–butyl–2,6–diformylphenol 2.062 g (10 mmol) in 20 mL of acetonitrile was added to the stirred solution of 1.142 g (10 mmol) (1*R*, 2*R*)–diaminocyclohexane in 15 mL of acetonitrile. The resulted yellow suspensions was stirred at 50 °C for 16 h, filtered out, washed with acetonitrile three times and dried to give 2.451 g of light yellow product (86%).

1.3. Metal-organic cage 2

Metal-organic cage **2** was synthesized as described previously.[1]. A solution of 0.199 g (9 mmol) $Zn(CH_3COO)_2{\cdot}2H_2O$ dissolved in 30 mL of methanol was added to the stirred suspension of the 3+3 macrocycle **1** (0.516 g, 6 mmol) in 70 mL of methanol. The mixture was refluxed for 2 h, cooled down and left to stand at room temperature overnight to give 0.315 g (55%) of yellow product.

1.4. Single Crystal Growth

MeOH@**2**: Single crystals of MeOH@**2** were obtained by slow evaporation of reaction solution in methanol overnight at room temperature. Single crystals of **2α** were obtained by heating MeOH@**2** at 80 °C under a vacuum for 12 h. Single crystals of **2β** were obtained by heating **2α** at 180 °C under a vacuum for 12 h.

1.5. Methods

1.5.1 Solution NMR: Solution ^{1}H NMR spectra were recorded at 400.13 MHz using a Bruker Avance 400 NMR spectrometer.

1.5.2 Thermogravimetric analysis: TGA analysis was carried out using a Q5000IR analyzer (TA instruments) with an automated vertical overhead thermobalance. The samples were heated at the rate of 10 °C /min using dry N_2 as the protective gas.

1.5.3 Differential scanning calorimetry (DSC): DSC measurements were carried out on a TA Q2000 (instrument with a Refrigerated Cooling System 90 and an autosampler) at 10 °C/min under an N_2 atmosphere. The DSC measurements can be used for determining the temperature at which a phase transformation occurs.

1.5.4 Powder X-ray Diffraction (PXRD): For screening, PXRD patterns were collected in transmission mode on samples held on thin Mylar film in aluminum well plates on a Panalytical Empyrean diffractometer, equipped with a high throughput screening XYZ stage, X-ray focusing mirror, and PIXcel detector, using Cu-Kα (λ = 1.541 Å) radiation. For indexing, samples were loaded into borosilicate glass capillaries, and PXRD patterns were recorded in transmission mode on a Panalytical Empyrean diffractometer, equipped with a sample spinner, X-ray focusing mirror, and PIXcel detector, using Cu-Kα (λ = 1.541 Å) radiation. PXRD patterns were refined with Pawley refinement in TOPAS Academic. Cryogenic PXRD patterns (30-100 K) were collected using a Bruker D8-Advance Bragg-Brentano geometry employing Cu-Kα1 Ge(111) lines focused radiation (1.54059 Å) equipped with Oxford Cryosystems PheniX (12K - 300K) diffractometer.

1.5.5 Single crystal X-ray diffraction (SC-XRD): SC-XRD data sets were measured on a Rigaku MicroMax-007 HF rotating anode diffractometer (Mo-Kα radiation, λ = 0.71073 Å, Kappa 4-circle goniometer, Rigaku Saturn724+ detector). Absorption corrections, using the multi-scan method, were performed with the program CrysAlisPro 1.171.40.45a.

1.5.6 Gas sorption: Nitrogen adsorption and desorption isotherms for all samples were collected at 77 K using an ASAP2020 volumetric adsorption analyzer (Micrometrics Instrument Corporation). Carbon dioxide and hydrogen isotherms were collected up to a pressure of 1200 mbar on a Micromeritics ASAP2020 at 77 K for hydrogen, or at 273 and 298 K for carbon dioxide. All samples were degassed at 80 °C for 15 hours under a dynamic vacuum (10−5 bar) before analysis.

A fully automated Sieverts apparatus (Autosorb-iQ2, Quantachrome Instruments) was used to perform the Ar isotherm at 87.3 K and the hydrogen cryogenic adsorption experiments. The calibration cell was an empty analysis carried out at the same temperature and pressure range of each experiment; corrections relating the sample volume and the nonlinearity of the adsorbate were made. Around 50 mg of each sample was activated at 80 °C for ***2α*** and at 180 °C for **2β** under vacuum for 12 h in order to remove any solvent molecules. A coupled cryocooler based on the Gifford−McMahon cycle was used to control the sample temperature. The cooling system permitted us to measure temperatures from 20 to 300 K with a temperature stability of <0.05 K.

1.5.7 Thermal desorption spectroscopy (TDS)

TDS experiments were carried out on an in-house designed device with about 2 mg of each sample. The sample holder is screwed tightly to a Cu block, which is surrounded by a heating spiral in the high vacuum chamber. The Cu block is connected to a flowing helium cryostat, allowing cooling below 20 K. All the samples were first loaded in the sample holder and activated under vacuum for 2 h at the temperature of 353 K for **2α** and at 453 K for **2β**. Then, the sample was exposed to a 10/200 mbar equimolar D_2/H_2 isotope mixture at different exposure temperatures (30, 50, 77, and 100 K) for 10 min. The remaining gas molecules were removed at the corresponding exposure temperatures until high vacuum was reached again. Afterwards, the sample was rapidly cooled down below 20 K. Then a linear heating ramp (0.1 K/s) was applied, the desorbing gas was continuously detected using a mass spectrometer (QMS), recognizing a pressure increase in the sample chamber when gas desorbs. The area under the desorption peak was proportional to the desorbing amount of gas, which can be quantified after careful calibration of the TDS apparatus.

A solid piece of a diluted Pd alloy $Pd_{95}Ce_5$ (~0.5 g) was used to calibrate the mass spectrometer signal. Before the calibration, the oxide layer of the alloy was removed by etching with aqua regia. Then the alloy was heated up to 600 K under a high vacuum to remove any hydrogen that might be absorbed during the etching procedure. Afterwards, it was exposed to 40 mbar pure H_2 or pure D_2 for 1.5–2.5 h at 350 K. As H and D were bound preferentially to the Cerium atoms at low exposure pressures, the alloy could be handled under ambient conditions for a short time. The alloy was weighed after being cooled down to room temperature. The mass difference between unloaded state and loaded state was equal to the mass uptake of hydrogen or deuterium, respectively. After weighing, the alloy was loaded in the chamber again, and then a 0.1 K/s heating ramp (room temperature (RT) to 600 K) was applied for a subsequent desorption spectrum. The obtained mass of gas is directly corresponded to the area under the desorption peak.

1.5.8 Computational details

An isolated molecule, extracted from experimental crystal structure of **2α**, was optimized by GFN2-XTB method with D4 dispersion[2] model in gas phase with defaults for convergence. The optimized geometry was confirmed as a true minima by numerical harmonic frequency calculation without imaginary frequency.[3] Based on that, MD simulation was performed for 200 ps, in which 100 ps for equilibration and 100 ps for production with timestep of 2 fs, along with SHAKE restraints on all bonds,[4] in the NVT ensemble using the Berendsen thermostat[5] to maintain temperature of 298 K. Structures were dumped every 1 ps. The results will be very similar by using isolated molecule from **2β**.

Pywindow[6] was used to calculate pore diameter and widow diameter of the molecular dynamics trajectories including 100 geometries obtained from xTB calculations.

Pore size distribution (PSD) histogram was calculated by *Zeo++*[7], it is advised to use probe radius similar to atomic radii for which one should expect the said 0.1 Å accuracy in peak positions. Therefore, we chose the probe radii below the half of the largest free sphere (D_f) of the crystals' pore structure. As for the MeOH@**2**, **2α** and **2β**, the D_f is 3.4, 2.0 and 2.6 Å, respectively. The probe radii chosen for MeOH@**2** and **2β** is 1.2 Å, and 0.97 Å is for **2α**.

1.5.9 Water stability measurements

5 mg of **2α** or **2β** was added to a 5 mL vial containing 4 mL of deionized water to test hydrolytic stability of **2α** and **2β**,. The **2α** and **2β** samples were suspended in water without stirring at RT for 1, 2, and 5 days. Then, each **2α** and **2β** sample was removed by filtration and dried in air. PXRD patterns were recorded using the air-dried samples (Figure S11), and NMR spectra were recorded after fully dissolving the air-dried samples in $CDCl_3$ (Figure S4 for **2α** and Figure S5 for **2β**). As shown in Figures S4 and S5, the ^{1}H NMR spectra recorded after fully dissolving the air-dried **2α** and **2β** crystals in $CDCl_3$ after being immersed in water for 1, 2, or 5 days are comparable, demonstrating that **2** is chemically stable in **2α** and **2β** in water at room temperature for at least 5 days

The PXRD patterns that were recorded for **2α** (Figure S11a) and **2β** (Figure S11b) after these samples were immersed in water for up to 5 days showed that some of the peaks shifted, and there were other differences in peak position. These differences indicate that **2α** and **2β** swell and change the structure slightly after being immersed in water. However, **2α** and **2β** remain crystalline, and their structures do not appear to collapse.

1.5.10 MeOH vapour sorption experiments

The large yellow block-shaped crystals of MeOH@**2** formed during synthesis immediately lose solvent after being removed from MeOH and break up into smaller yellow crystalline powders in the air. The PXRD pattern of the air-dried MeOH@**2** sample is not identical to the simulated PXRD pattern of MeOH@**2**; however, it closely matches the simulated PXRD pattern of the **2α** structure (Figure S12).

To further investigate the dynamic structural behaviour of **2α** and **2β**, we performed vapour sorption experiments using pure MeOH. For each MeOH vapour exposure test, an open 5 mL vial containing 15 mg of **2α** or **2β** was placed in a sealed 20 mL vial containing 2 mL of MeOH at RT. PXRD patterns of the MeOH-loaded samples were then collected for up to 5 days immediately after air-drying the samples on the PXRD plate. The PXRD pattern of **2α** after being exposed to MeOH vapour for 2 days is similar to the PXRD of air-dried MeOH@**2** (Figure S12a). By contrast, the PXRD patterns of **2β** after being exposed to MeOH vapour appears to gradually transform into PXRD of air-dried MeOH@**2** over the 5-days before the crystals begin to redissolve in MeOH in the vial (Figure 12b).

2. Crystallography Report

Table S1. SC-XRD data for MeOH@**2**, **2α** and **2β**.

Molecule	MeOH@**2**	**2α**	**2β**
Crystallisation Solvent	MeOH	-	-
Space Group	*Cc*	*P1*	*P1*
Wavelength [Å]	Mo-Kα	Mo-Kα	Mo-Kα
Collection Temperature	100 K	100 K	100 K
Formula	$C_{108}H_{138}N_{12}O_7$ Zn_3, 10.5(CH_3OH), 3.25(H_2O)	2($C_{108}H_{138}N_{12}O_6Zn_3$), 3.25($H_2O$)	$C_{108}H_{138}N_{12}O_6Zn_3$, 0.5($H_2O$)
Mr	2307.40	3851.36	1905.94
Crystal Size (mm)	0.213×0.159 ×0.159	0.123×0.121×0.079	0.133×0.124×0.111
Crystal System	monoclinic	triclinic	triclinic
a [Å]	18.9478(9)	14.6670(10)	14.1697(11)
b [Å]	24.3539(9)	18.0763(12)	14.4152(13)
c [Å]	15.6901(11)	24.2155(14)	16.3546(16)
α [°]		78.958(5)	108.741(9)
β [°]	111.865(7)	73.662(5)	94.396(7)
γ [°]		68.325(6)	109.934(8)
V [$Å^3$]	6719.4(7)	5697.6(7)	2908.2(5)
Z	2	1	1
D_{calcd} [g cm^{-3}]	1.140	1.122	1.088
μ [mm^{-1}]	0.594	0.681	0.666
F(000)	2475	2048	1013
2θ range [°]	3.34 – 46.51	3.19 – 46.51	3.67 – 49.42
Reflections collected	36167	46181	38781
Independent reflections, R_{int}	9057, 0.0723	27140, 0.1176	17131, 0.1029
Obs. Data [$I > 2\sigma$]	7068	14768	10409
Data / restraints / parameters	9057/97/717	27140/2396/2412	17131/105/1150
Final R_1 values ($I > 2\sigma(I)$)	0.0745	0.0814	0.0836
Final R_1 values (all data)	0.0989	0.1733	0.1533
Final $wR(F^2)$ values (all data)	0.1975	0.1474	0.1620
Goodness-of-fit on F^2	1.039	1.001	1.028
Largest difference peak and hole [e.A^{-3}]	0.563 /-0.374	0.689/-0.442	0.987/-0.510
CCDC	2151938	2151939	2151940

3. Characterization

3.1 NMR Spectra

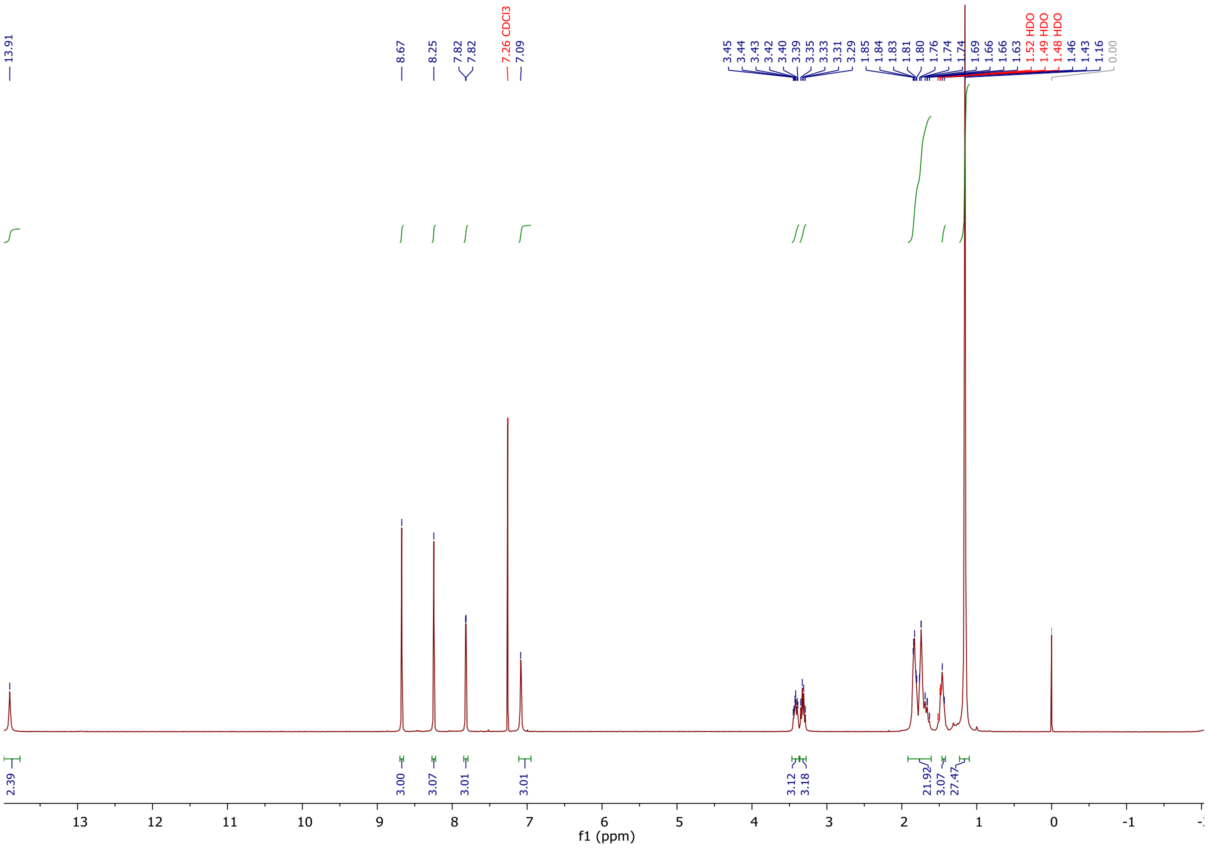

Figure S1. ^{1}H NMR spectrum (400 MHz, $CDCl_3$, 293 K) of macrocycle **1**-(*R,R*) ($C_{54}H_{72}N_6O_3$): δ_H 13.91 (s,OH, 3H), 8.67 (s, CH=N, 3H), 8.25 (s, CH=N, 3H), 7.82 (d, ArH, 3H), 7.09 (br, s, ArH, 3H), 3.42 (m, CHN, 3H), 3.34 (q, CHN, 3H), 1.43-1. 85 (m, CH_2, 24H), 1.19 (s, CH_3, 27H). The ^{1}H NMR spectrum of **1** is consistent with literature reported data.[1]

^{1}H NMR spectrums (400 MHz, $CDCl_3$, 293 K) of **2*α*** and **2*β*** ($C_{108}H_{138}N_{12}O_6Zn_3$) are shown in Figure S2–S3: 9.49 (s, CH=N, 6H), 8.06 (s, CH=N, 6H), 7.86 (d, ArH, 6H), 6.98 (d, ArH, 6H), 3.77 (m, CHN, 6H), 3.32, (m, CHN, 6H), 1.27-1.85 (m, CH_2, 42H), 1.20 (s, CH_3, 54H), 0.89 (m, CH_2, 6H). The ^{1}H NMR spectrum of **2** is consistent with literature reported data.[1]

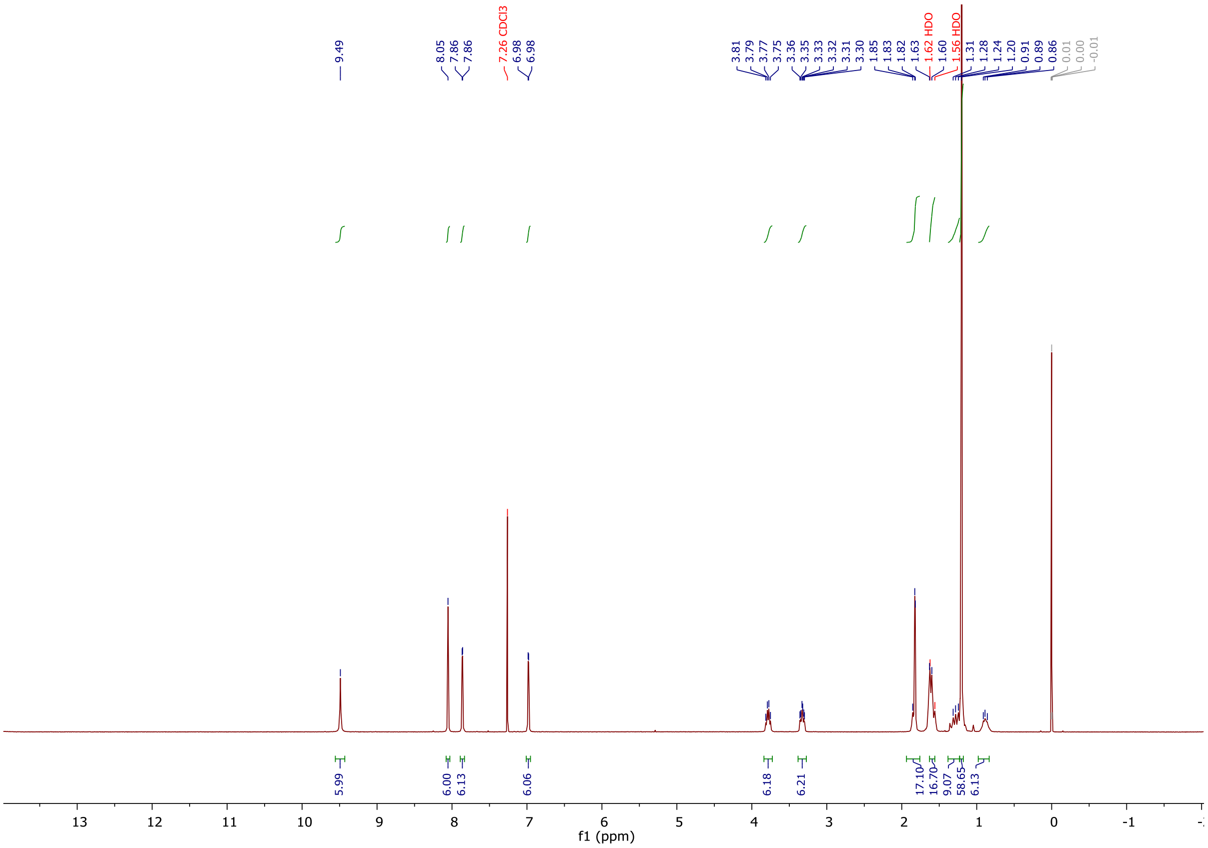

Figure S2. ^{1}H NMR spectrum (400 MHz, $CDCl_3$, 293 K) of **2α** activated at 80 °C. There is no signal for the solvent-methanol in the spectrum, indicating that the methanol in **2** could be removed at 80 °C.

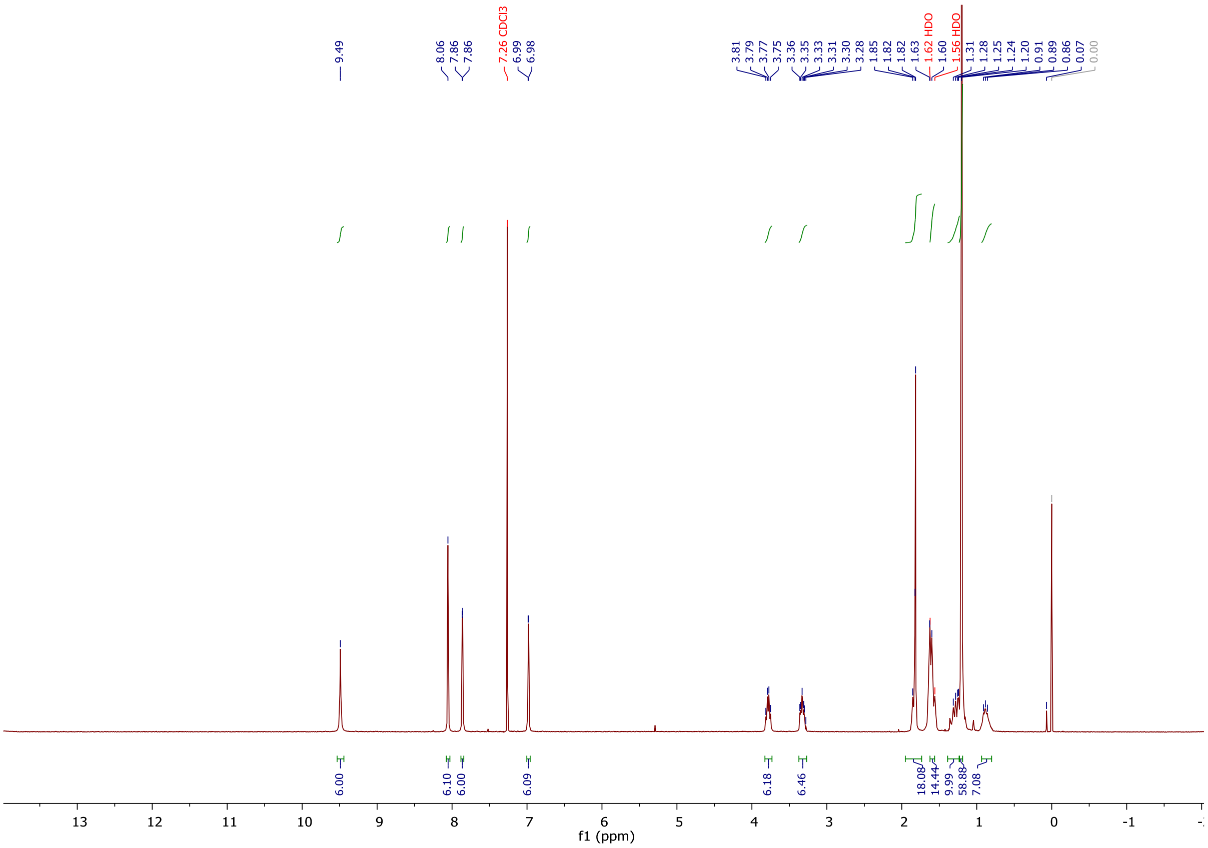

Figure S3. ^1H NMR spectrum (400 MHz, $CDCl_3$, 293 K) of **2β** activated at 180 °C.

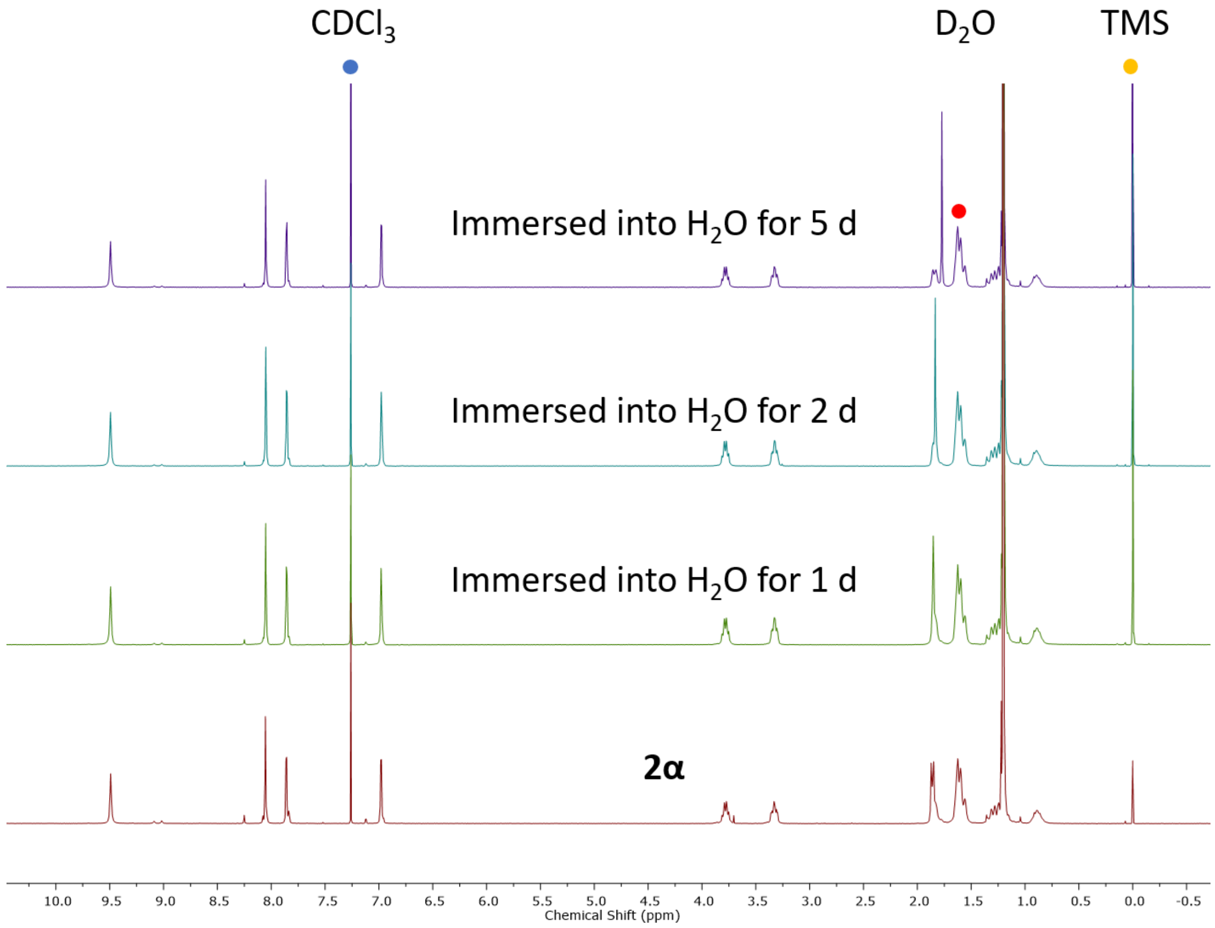

Figure S4. ^{1}H NMR spectrum (400 MHz, $CDCl_3$, 293 K) of **2α** after being suspended in water at RT.

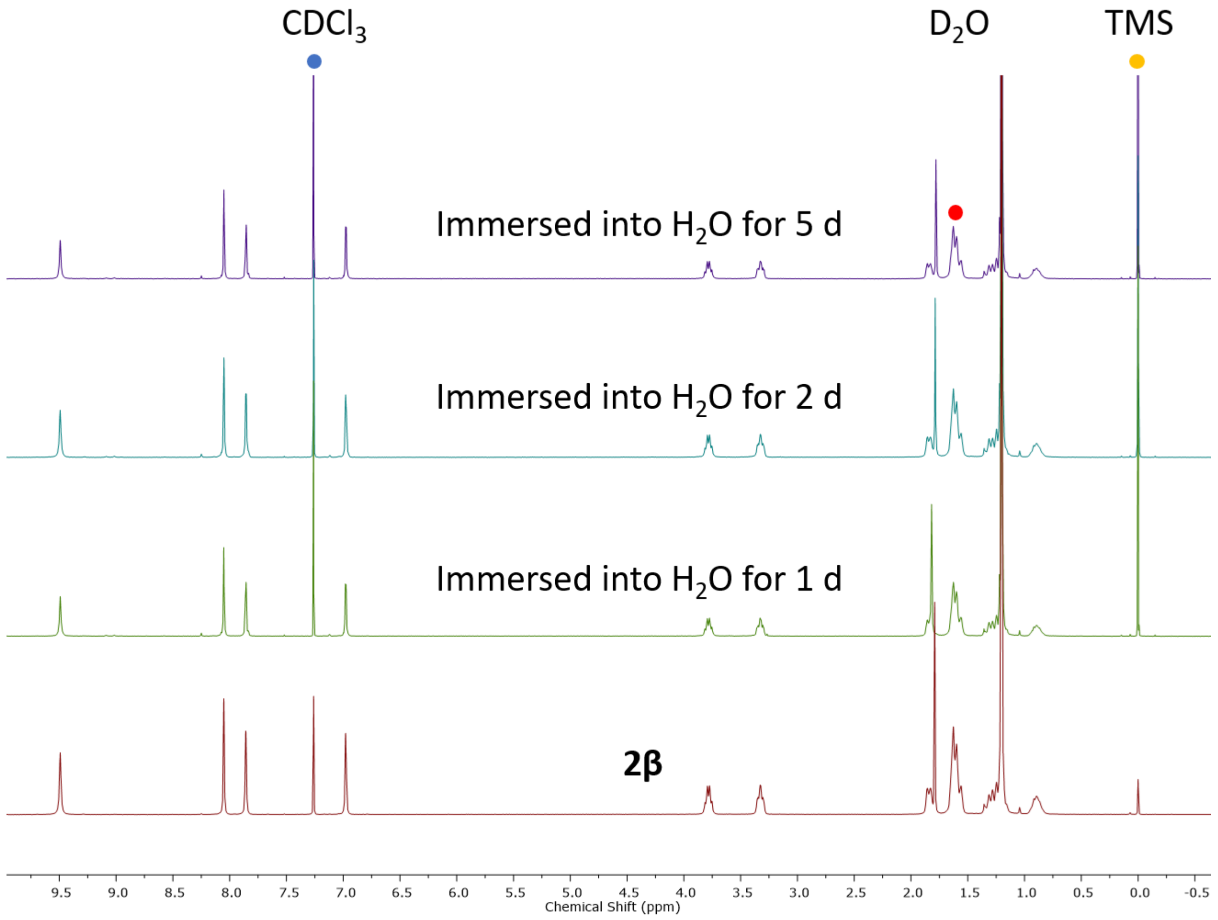

Figure S5. ^{1}H NMR spectrum (400 MHz, $CDCl_3$, 293 K) of **2β** after being suspended in water at RT.

3.2 TGA Data

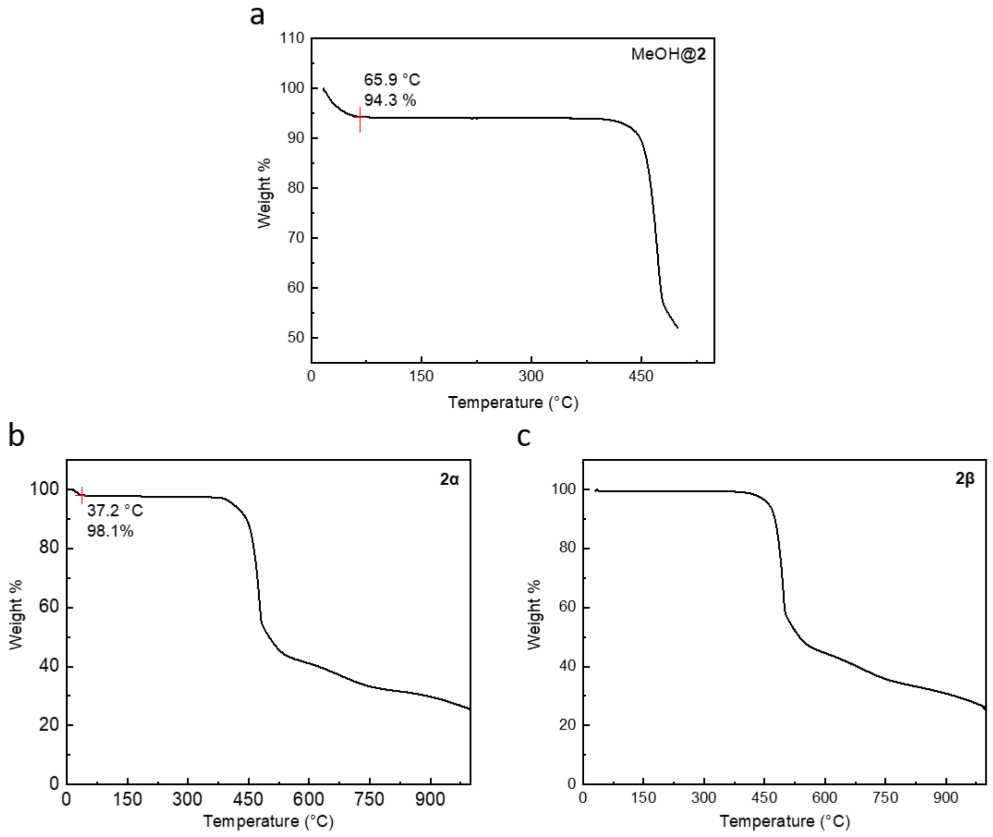

Figure S6. Thermogravimetric analyses of metal-organic cage **2**: (a) MeOH@**2**, (b) **2α** activated at 80 °C, (c) **2β** activated at 180 °C. There is a distinct solvent lost in MeOH@**2** at 65.9 °C, as shown in (a), which is consistent with the boiling point of methanol (64.7 °C). As for **2α** shown in (b), there is little weight loss at 37.2 °C may be due to the unbound water, which is consistent to the NMR result that the methanol has been removed entirely in **2α**. (Figure S2) A temperature of ⩾80 °C can remove all of the methanol in **2**.

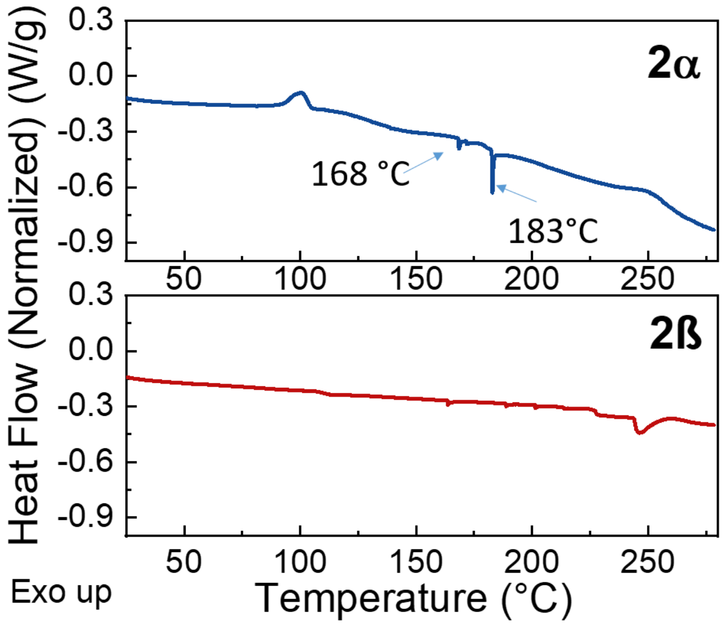

Figure S7. DSC curves of **2α** and **2β** heated at a ramp rate of 10 °C/min under an N_2 atmosphere.

3.3 PXRD Data

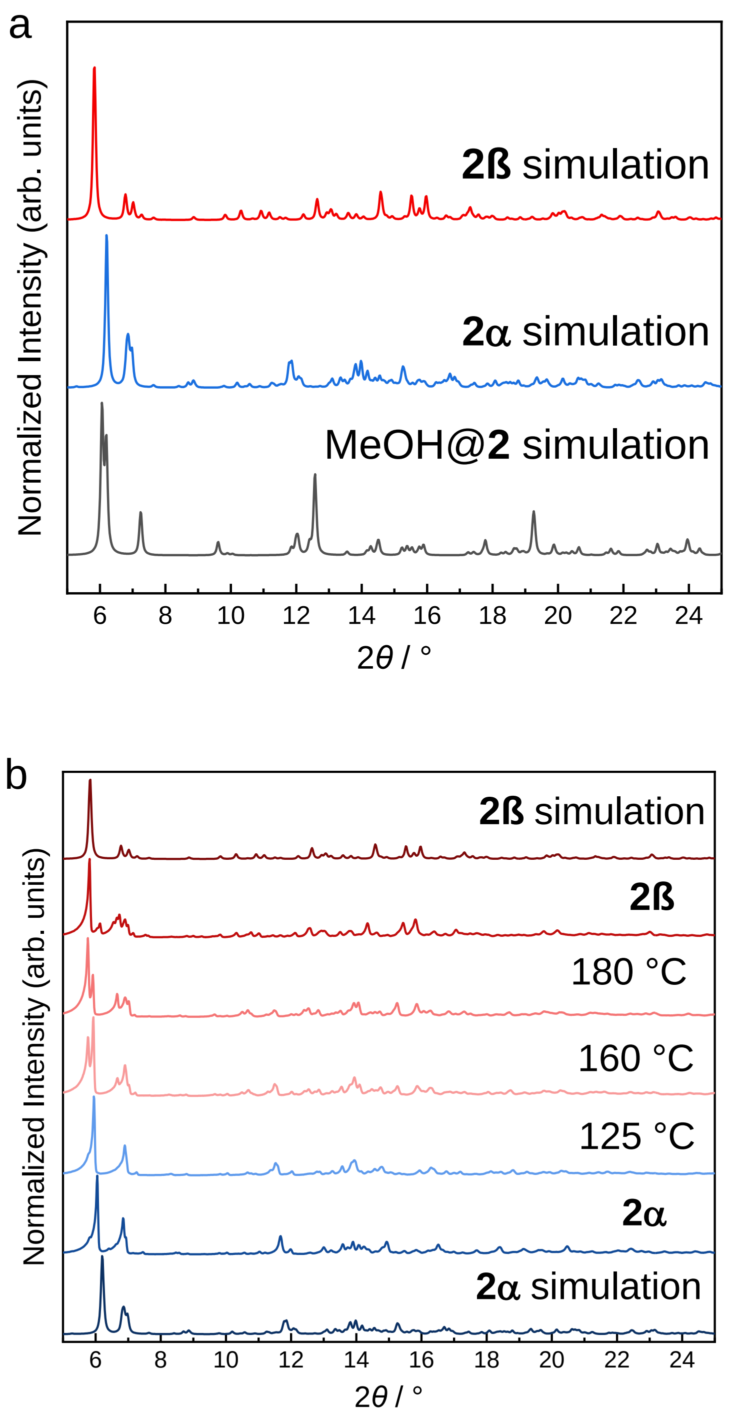

Figure S8. (a) Simulated PXRD pattern for MeOH@**2**, **2α** and **2β** using the single crystal structures. (b) PXRD patterns that were collected during *in situ* heating **2α** from 30 to 180 °C and simulated PXRD patterns for **2α** and **2β**.

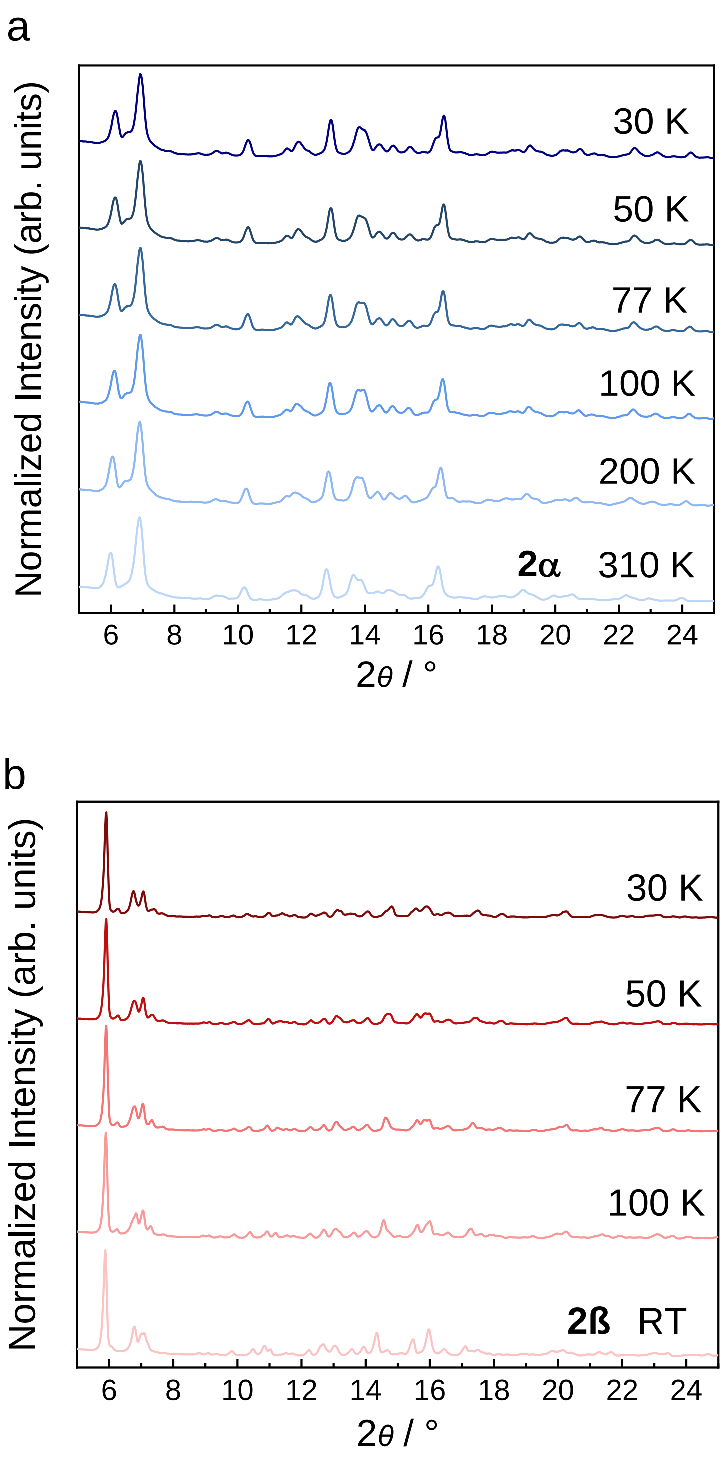

Figure S9. Variable-temperature powder X-ray diffraction (PXRD) patterns: (a) PXRD patterns of **2α** collected by heating from 30 to 310 K. (b) PXRD patterns of **2β** collected by heating from 30 to 298 K. The relative peak positions and intensities of VT-PXRD patterns collected under vacuum at cryogenic temperature (30–100 K) are slightly different to the PXRD patterns collected in the air at room temperature. We ascribe these slight differences to the different measurement conditions (pressure, temperature, humidity). However, the VT-PXRD patterns closely match the other PXRD patterns collected during this study and the simulated PXRD pattern of the SXRD structure, indicating the crystal structure is comparable.

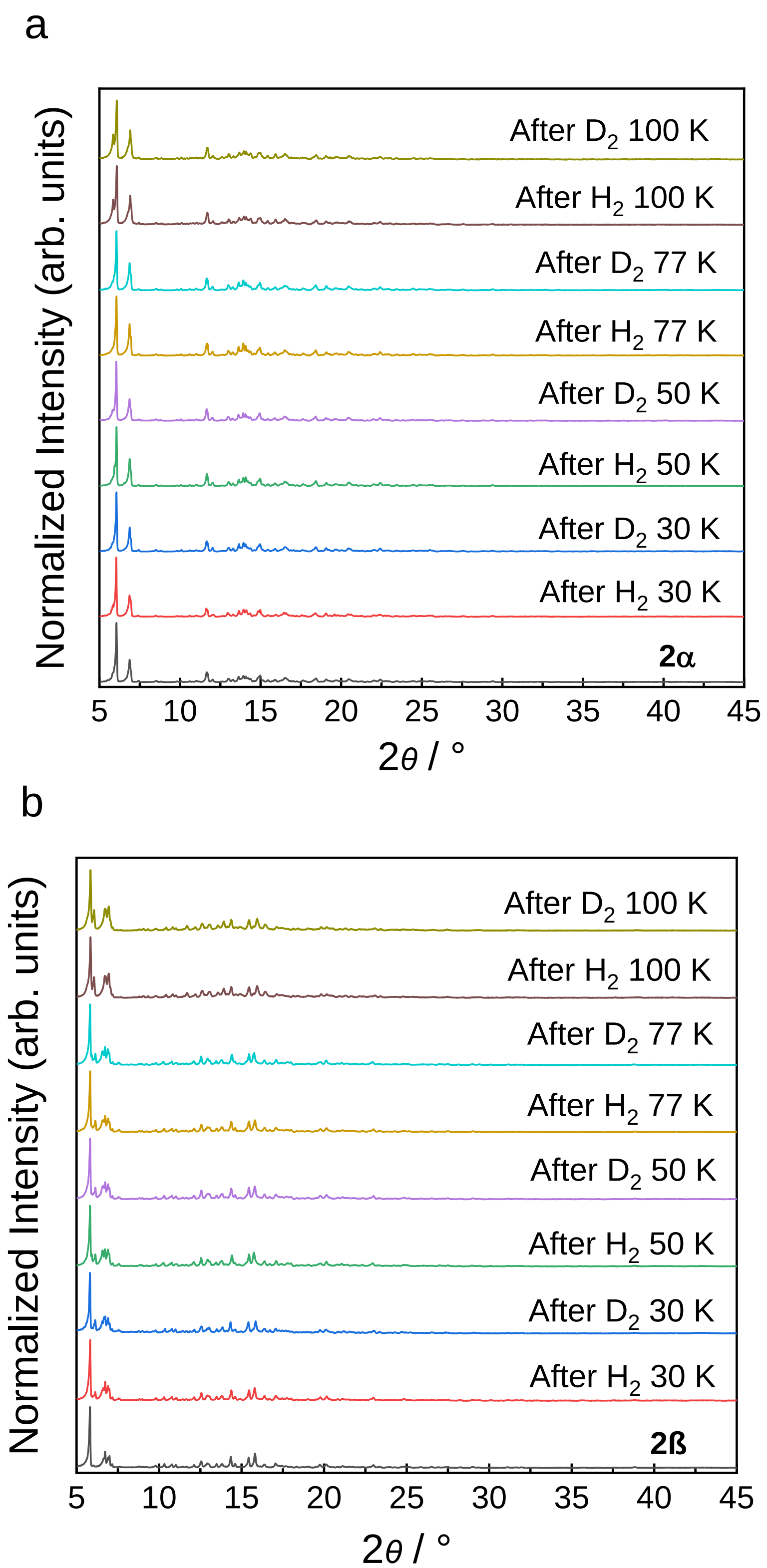

Figure S10. PXRD patterns of **2** after and before testing gas isotherms: (a) PXRD patterns of **2α** collected after and before testing H_2 and D_2 isotherms at 30 to 100 K. (b) PXRD patterns of **2β** collected after and before testing H_2 and D_2 isotherms at 30 to 100 K.

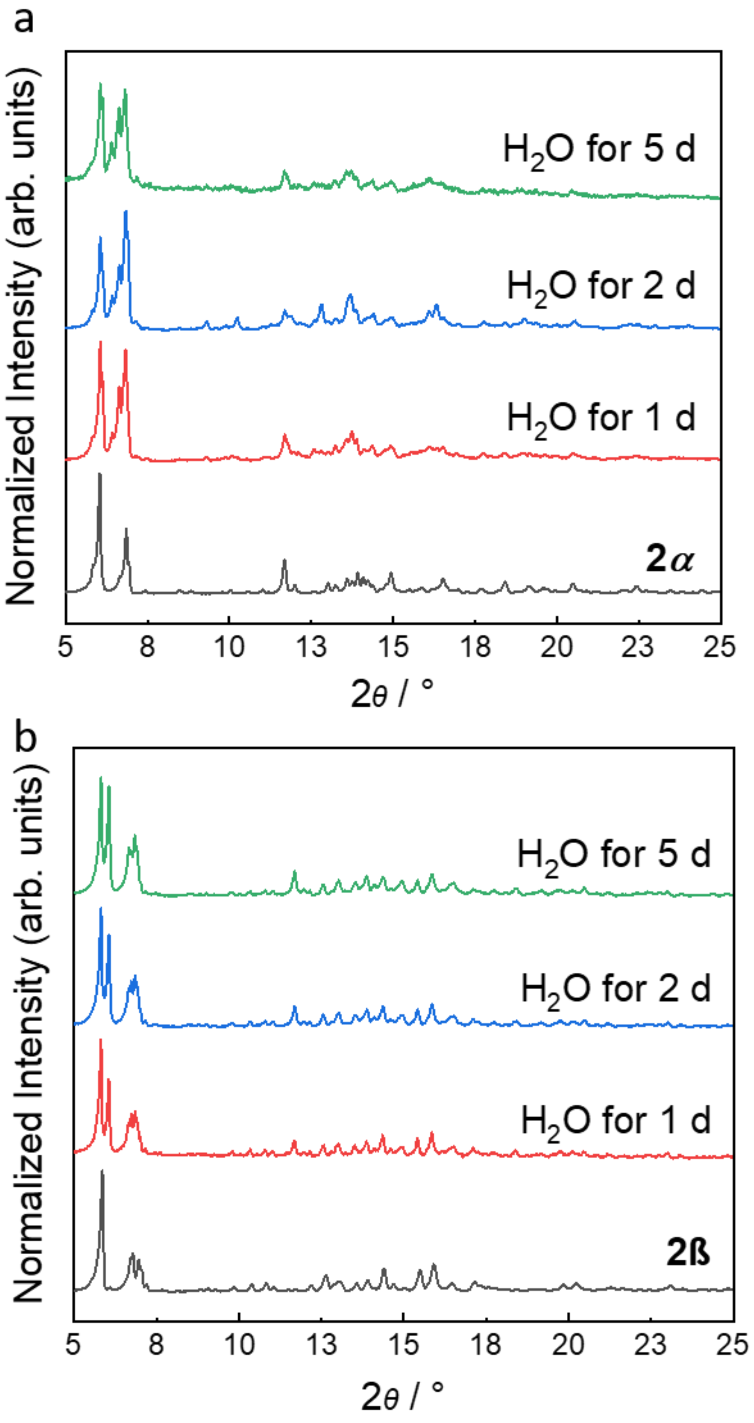

Figure S11. PXRD patterns that were collected after suspending (a) **2α** in water and (b) **2β** in water for 1, 2, and 5 days. The samples were collected by filtration and air-dried before the PXRD patterns were recorded.

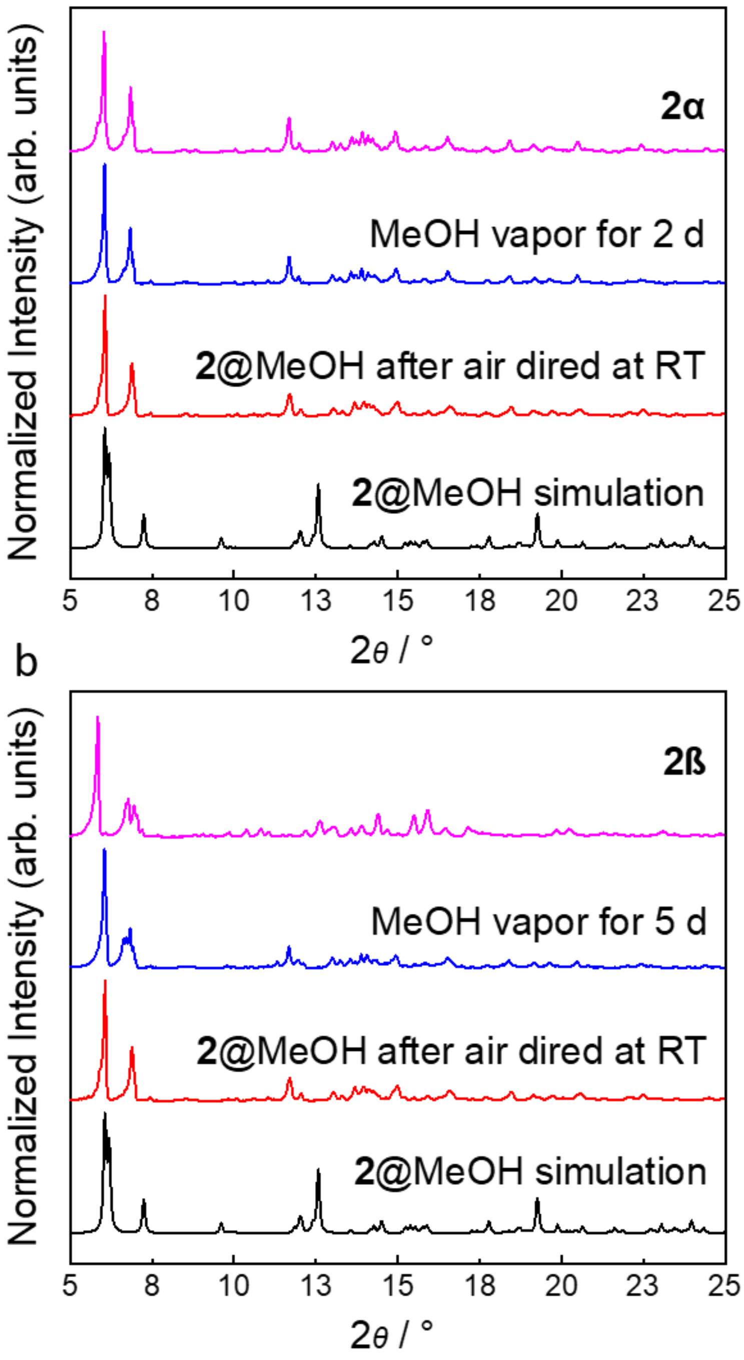

Figure S12. PXRD patterns of (a) **2α** and (b) **2β** after being exposed to MeOH vapour at RT for up to 5 d. The PXRDs of 2@MeOH after air-drying the sample at RT on the PXRD plate (red) and the simulated PXRD of MeOH@2 (black) are included in both plots.

3.5. Gas Sorption Analysis

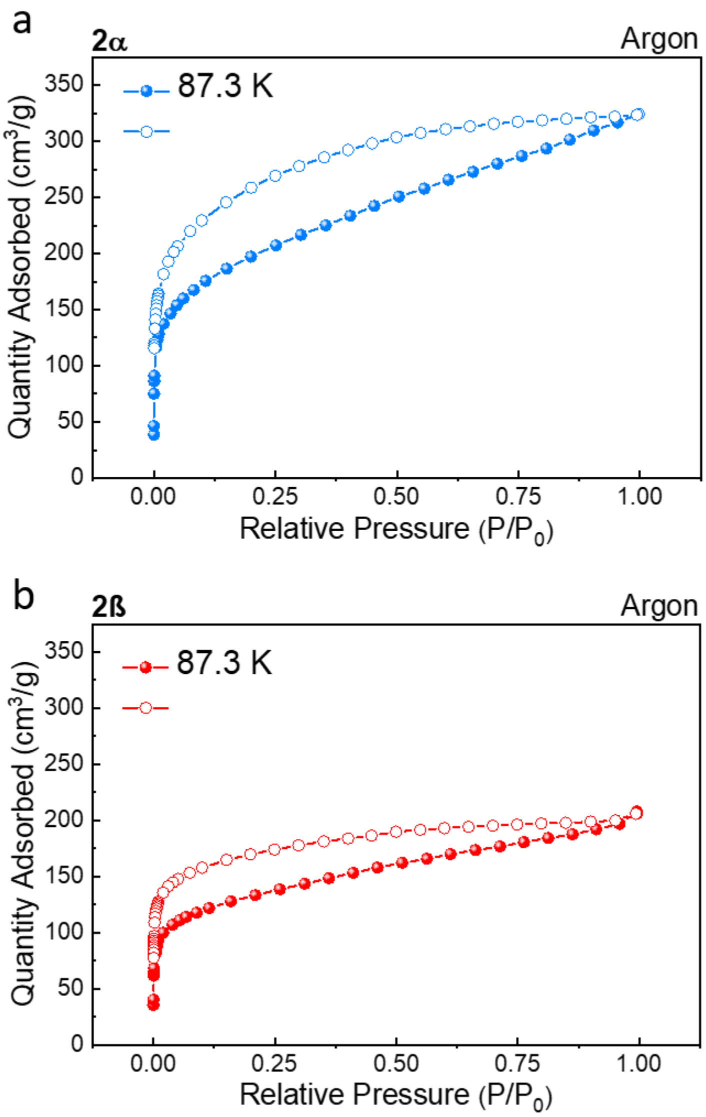

Figure S13. Argon (Ar) sorption isotherms at 87.3 K for (a) **2*α*** (blue) and (b) **2*β*** (red). Solid symbols: adsorption; hollow symbols: desorption.

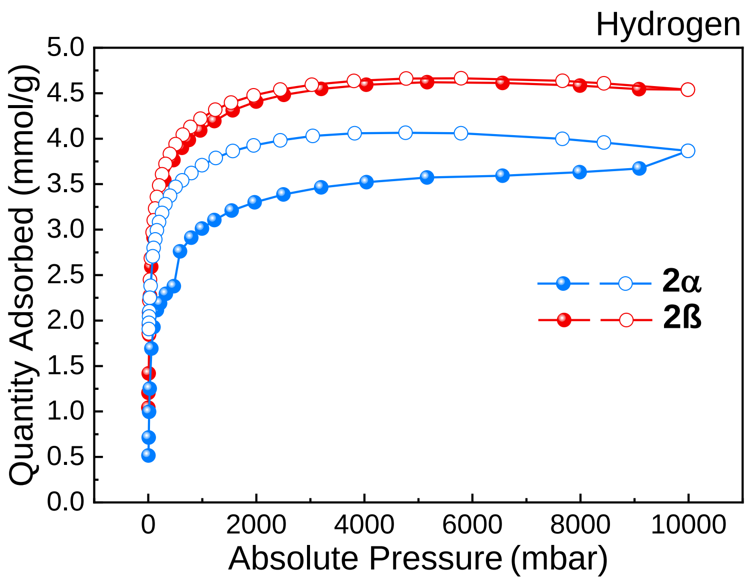

Figure S14. H_2 isotherms of **2α** (blue) and **2β** (red) at 77 K from 0 to 10 bar. Solid symbols: adsorption; hollow symbols: desorption.

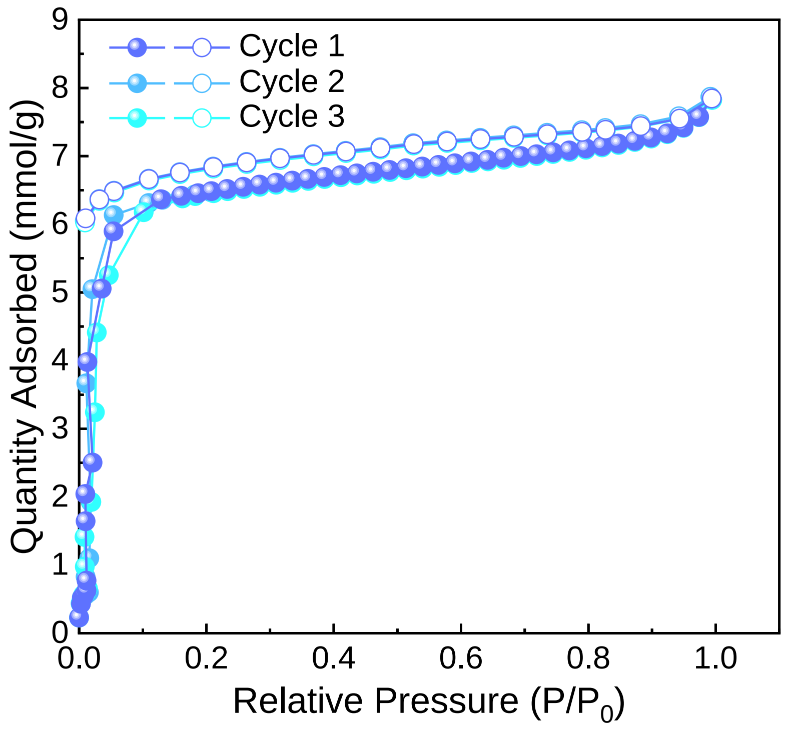

Figure S15. N_2 adsorption-desorption cycles of **2α** recorded at 77 K. Solid symbols: adsorption; hollow symbols: desorption. The isotherms were cycled to investigate the reproducibility of the pressure-induced gating effect observed for **2α** during N_2 adsorption at 77 K. The N_2 adsorption-desorption isotherm three times in a row using the same **2α** sample. The three N_2 adsorption isotherms all exhibited pressure-induced gating effects over the P/P_0 range from 0.01 to 0.12, and have similar shapes and uptakes outside that range. In addition, the calculated BET surface areas for cycles 1, 2, and 3 were comparable (401, 418, and 398 m^2/g, respectively) over the P/P_0 range (0.06 to 0.32).

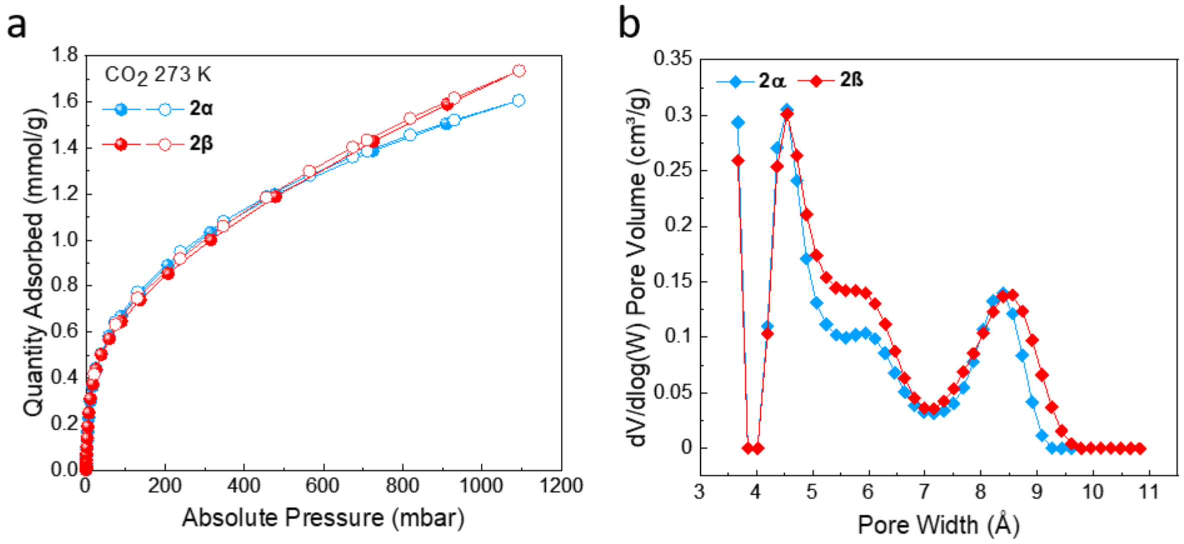

Figure S16. (a) CO_2 isotherms at 273K for **2α** and **2β**. Solid symbols: adsorption; hollow symbols: desorption. (b) Pore size distributions of **2α** and **2β** calculated by the CO_2 DFT model (Micromeritics). The calculated pore size distributions of **2α** and **2β** are similar – around 3.7 to 9.6 Å. The pore size distributions calculated based on the CO_2 isotherm cover a larger pore size range than that calculated based on idealised crystal structure where the pore sizes of **2α** and **2β** centre mainly around 2.0 to 5.1 Å and 2.6 to 4.1 Å .

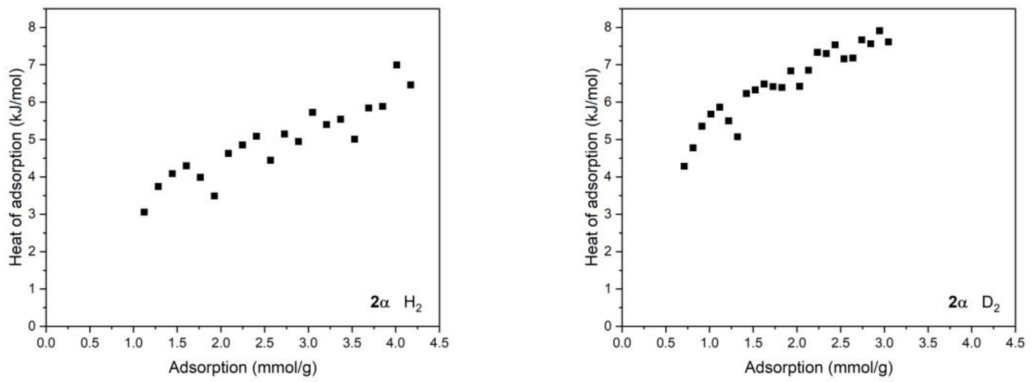

Figure S17. Isosteric heat of adsorption of H_2 and D_2 for **2α** as a function of the adsorption amount. The isosteric heat of adsorption in **2α** was calculated to be 3–7 kJ/mol for H_2 and 4–8 kJ/mol for D_2 using the Clausius-Clapeyron equation. A higher heat of adsorption is the reason for the higher D_2 uptake. Interestingly, the enthalpy gradually rises with a higher adsorption amount, meaning that the host can adsorb gas molecules more easily at higher loadings. Meanwhile, the heat of adsorption in **2β** was calculated to be 0.5–3.5 kJ/mol for both isotopes. The abnormal slope for **2α** is attributed to the strong diffusion barrier due to the small pore aperture, which opens up at higher temperatures due to thermally-activated flexibility.

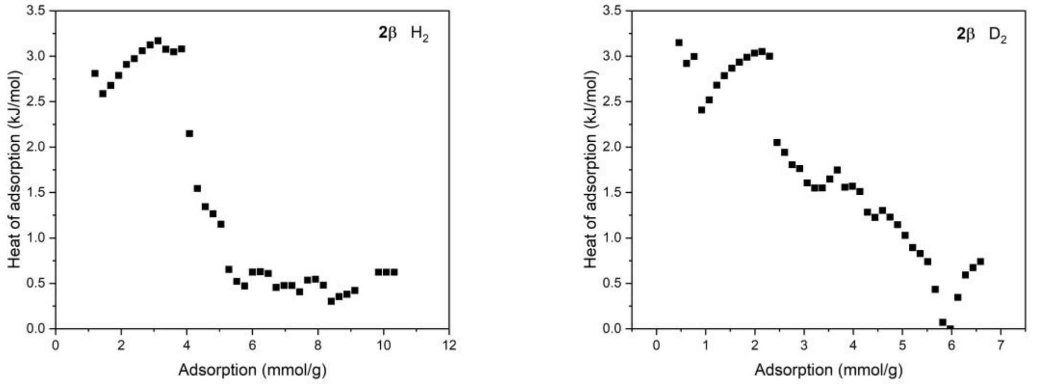

Figure S18. Isosteric heat of adsorption of H_2 and D_2 for **2β** as a function of the adsorption amount. Concerning the self-diffusion coefficient, for materials like MOCs, the diffusion limitation typically governs the adsorption/ desorption process. At low temperatures, gas molecules can only be adsorbed weakly on the outer surface; the gas can penetrate into the cavity at higher exposure temperatures. It is, therefore, difficult to calculate the self-diffusion coefficient for hydrogen isotopes. However, the maximum temperature of the TDS peaks correlates with the diffusion thru the apertures; that is, lower and higher maximum temperatures correspond to faster and slower diffusion, respectively. Thus, the higher maximum temperature of H_2 (106 K **2α** in and 92 K in **2β**) compared to D_2 (102 K **2α** in and 86 K in **2β**) indicates a slower H_2 and faster D_2 diffusion in MOC.

3.6 TDS

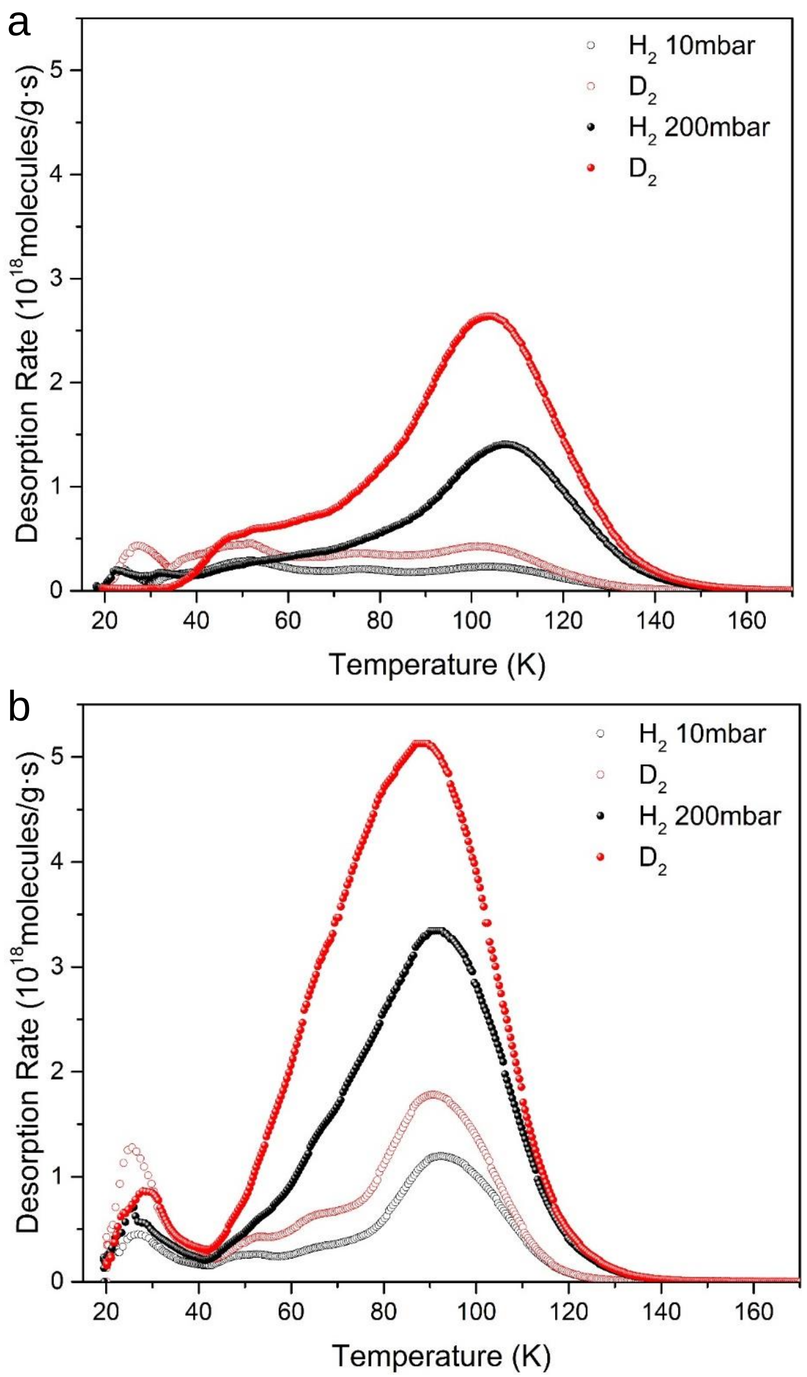

Figure S19. H_2 (Black) and D_2 (Red) thermal desorption spectra of H_2/D_2 single gas at 10 mbar and 200 mbar for (a) **2α** and (b) **2β**. A laboratory-designed cryogenic thermal-desorption spectroscope (TDS) are utilized for determining the preferred H_2 and D_2 adsorption sites in **2α** and **2β**. TDS measurements were carried out by applying pure H_2 and D_2 atmospheres (10/200 mbar), respectively, under identical experimental conditions. The gas exposure was carried out at room temperature and cooling down to 20 K. The resulting TDS spectra obtained between 20 and 170 K.

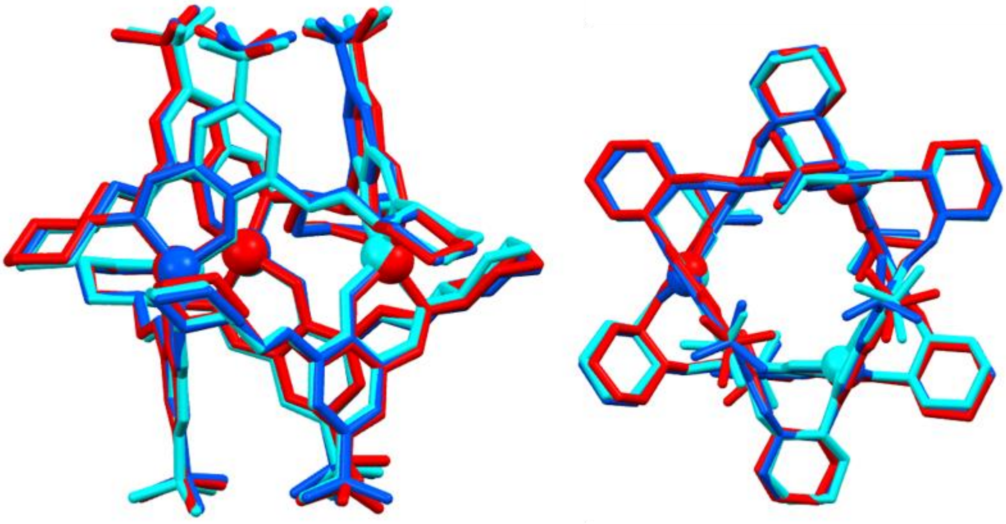

Figure S20. Molecules overlay for **2α** (blue and cyan) and **2β** (red), as generated using the Molecules Overlay tool in Mercury. H atoms are omitted for clarity.

Table S2. Summary of hydrogen isotope KQS selectivities above 77 K with various porous materials without open metal sites.

Compound	Aperture (Å)	T (K)	P (mbar)	Selectivity (D_2/H_2) (1:1 Mixture)	Ratio (nD_2/nH_2) (Pure Gas Isotherms)	Ratio (kD_2/kH_2) (Rate constant)	Ref.
CMS	5-7/ 15-35	77	4000	1.53 (H_2:D_2=139:175)			[8]
Zeolite 5A	5	77		1.8 (H_2:D_2=99:1)			[9]
Zeolite 13X	8	77		1.9 (H_2:D_2=99:1)			
Zeolite Y	6-7	77	4000	Up to 1.52 (H_2:D_2=139:175)			[10]
CMS T3A	5.46	77	5-1000		1.063		[11]
PCS	5.66	77	50-500		-	Up to 1.9	
		77	5-1000		1.097	-	
		77	0-50			Up to 1.25	
3KT-172	4.9	77	20			1.86	[12]
1.5GN-H	4.6	77	100			5.83	
CNH	-	77	70-1000		1.09		[13]
HKUST-1	9/5	77	20		1.23		
SWCNT	13-14	77	10000		1.2		[14]
SG-SWCNT	28.5	77	0.1-10	Up to 3.8*			
LA-SWCNT	13.7	77	0.1-10	Up to 1.5*			
Zeolite 5A	5	77	0.01-10	Up to 3.26*			[15]
Zeolite NaX	7.4	77	139		1.18		[16]
			STP		1.33		
Zeolite 4A	4	77	150	2.09*			[17]
Zeolite 5A	5	77	150	2.48*			
Zeolite Y	6-7	77	150	1.32*			
Zeolite 10X	8	77	150	1.3*			
CMK-3	35	77	150	0.95*			
Zeolite 13X	8	77	0.1-1000	Up to 3.2*			[18]
			0.1-10	Up to 5.8*			
			50-1000		1.13		
M'MOF 1	5.6×12	77	5-1000		1.09		[19]
		87			1.11		
VSB-5	11	140	0-1000	Up to 4*			[20]
12-Connected MOFs	10	77	1000		1.1		[21]
Cu_2L_2	7.3	77	100		1.2		[22]
FMOFCu	2.5, 3.6	77	50	3-4			[23]
		87	50	3-4			
IFP-4	1.7	77	60	2.1			[24]
IFP-7	2.1	77	60	1.5			

ZIF-67	3.4	77	STP	1.29			[25]
ZIF-67 @NH_2-γ-Al_2O_3	3.4	77	STP	1.79			
ZIF-67 @NH_2–SiO_2	3.4	77		1.52			[26]
FJI-Y11	3.8, 8.4	77		1.76			[27]

* D_2/H_2 mixture selectivity calculated by IAST

References

[1] A. Sarnicka, P. Starynowicz, J. Lisowski, *Chem. Commun.* **2012**, *48*, 2237-2239.

[2] E. Caldeweyher, S. Ehlert, A. Hansen, H. Neugebauer, S. Spicher, C. Bannwarth, S. Grimme, *J. Chem. Phys.* **2019**, *150*, 154122.

[3] a) C. Bannwarth, E. Caldeweyher, S. Ehlert, A. Hansen, P. Pracht, J. Seibert, S. Spicher, S. Grimme, *WIREs Comput. Mol. Sci.* **2021**, *11*, e1493; b) S. Grimme, C. Bannwarth, P. Shushkov, *J. Chem. Theory Comput.* **2017**, *13*, 1989-2009; c) C. Bannwarth, S. Ehlert, S. Grimme, B. P. S.-C. Tight-Binding, *J. Chem. Theory Comput.* **2019**, *15*, 1652-1671.

[4] J.-P. Ryckaert, G. Ciccotti, H. J. C. Berendsen, *J. Comput. Phys.* **1977**, *23*, 327-341.

[5] H. J. C. Berendsen, J. P. M. Postma, W. F. v. Gunsteren, A. DiNola, J. R. Haak, *J. Chem. Phys.* **1984**, *81*, 3684-3690.

[6] M. Miklitz, K. E. Jelfs, *J. Chem. Inf. Model.* **2018**, *58*, 2387-2391.

[7] M. Pinheiro, R. L. Martin, C. H. Rycroft, A. Jones, E. Iglesia, M. Haranczyk, *J. Mol. Graphics Modell.* **2013**, *44*, 208-219.

[8] X.-Z. Chu, Z.-P. Cheng, Y.-J. Zhao, J.-M. Xu, M.-S. Li, L. Zhou, C.-H. Lee, *Sep. Purif. Technol.* **2015**, *146*, 168-175.

[9] A. J. Arvai, C. Nielsen, Area Detectir System Corporation, Poway, CA, USA, **1983**.

[10] X.-Z. Chu, Y.-J. Zhao, Y.-H. Kan, W.-G. Zhang, S.-Y. Zhou, Y.-P. Zhou, L. Zhou, *Chem Eng J* **2009**, *152*, 428-433.

[11] X. Zhao, S. Villar-Rodil, A. J. Fletcher, K. M. Thomas, *J. Phys. Chem. B* **2006**, *110*, 9947-9955.

[12] Y. Xing, J. Cai, L. Li, M. Yang, X. Zhao, *PCCP* **2014**, *16*, 15800-15805.

[13] H. Tanaka, H. Kanoh, M. Yudasaka, S. Iijima, K. Kaneko, *J. Am. Chem. Soc.* **2005**, *127*, 7511-7516.

[14] D. Noguchi, Y. Hattori, C. Yang, Y. Tao, T. Konishi, T. Fujikawa, T. Ohkubo, Y. Nobuhara, T. Ohba, H. Tanaka, *ECS Trans.* **2007**, *11*, 63-75.

[15] K. Kotoh, T. Nishikawa, Y. Kashio, *J. Nucl. Sci. Technol.* **2002**, *39*, 435-441.

[16] J. Salazar, S. Lectez, C. Gauvin, M. Macaud, J. Bellat, G. Weber, I. Bezverkhyy, J. Simon, *Int. J. Hydrog. Energ.* **2017**, *42*, 13099-13110.

[17] X.-Z. Chu, Y.-P. Zhou, Y.-Z. Zhang, W. Su, Y. Sun, L. Zhou, *J. Phys. Chem. B* **2006**, *110*, 22596-22600.

[18] K. Kotoh, K. Kudo, *Fusion Sci. Technol.* **2005**, *48*, 148-151.

[19] B. Chen, X. Zhao, A. Putkham, K. Hong, E. B. Lobkovsky, E. J. Hurtado, A. J. Fletcher, K. M. Thomas, *J. Am. Chem. Soc.* **2008**, *130*, 6411-6423.

[20] A. Sharma, K. V. Lawler, J. J. Wolffis, C. T. Eckdahl, C. S. McDonald, J. L. Rowsell, S. A. FitzGerald, P. M. Forster, *Langmuir* **2017**, *33*, 14586-14591.

[21] J. Jia, X. Lin, C. Wilson, A. J. Blake, N. R. Champness, P. Hubberstey, G. Walker, E. J. Cussen, M. Schröder, *Chem. Commun.* **2007**, 840-842.

[22] X. Lin, J. Jia, X. Zhao, K. M. Thomas, A. J. Blake, G. S. Walker, N. R. Champness, P. Hubberstey, M. Schröder, *Angew. Chem. Int. Ed.* **2006**, *45*, 7358-7364.

[23] L. Zhang, S. Jee, J. Park, M. Jung, D. Wallacher, A. Franz, W. Lee, M. Yoon, K. Choi, M. Hirscher, H. Oh, *J. Am. Chem. Soc.* **2019**, *141*, 19850-19858.

[24] S. S. Mondal, A. Kreuzer, K. Behrens, G. Schütz, H. J. Holdt, M. Hirscher, *Chemphyschem* **2019**, *20*, 1311.

[25] E. Ping, H. Ji, L. Zhang, Y. Zhou, C. Ding, X. Chen, *ACS Appl. Energy Mater.* **2021**, *4*, 10857-10866.

[26] X. Chen, M. Liu, L. Zhang, Y. Zhou, E. Ping, C. Ding, *Int. J. Hydrog. Energ.* **2021**, *46*, 13029-13037.

[27] Y. Si, X. He, J. Jiang, Z. Duan, W. Wang, D. Yuan, *Nano Res.* **2021**, *14*, 518-525.
